# Diastereoselective Synthesis of Highly Functionalized Proline Derivatives

**DOI:** 10.3390/molecules27206898

**Published:** 2022-10-14

**Authors:** Anna N. Philippova, Daria V. Vorobyeva, Pavel S. Gribanov, Fedor M. Dolgushin, Sergey N. Osipov

**Affiliations:** 1 A. N. Nesmeyanov Institute of Organoelement Compounds, Russian Academy of Sciences, Vavilov str. 28/1, 119334 Moscow, Russia; 2 N. S. Kurnakov Institute of General and Inorganic Chemistry, Russian Academy of Sciences, 31 Leninsky Prosp., 119071 Moscow, Russia; 3 Plekhanov Russian University of Economics, 36 Stremyanny per., 117997 Moscow, Russia

**Keywords:** 1,6-allenynes, cycloaddition, alder-ene reaction, catalysis, prolines, 1,2,3-triazoles

## Abstract

An efficient way to access highly functionalized proline derivatives was developed based on a Cu(I)-catalyzed reaction between CF_3_-substituted allenynes and tosylazide, which involved a cascade of [3 + 2]-cycloaddition/ketenimine and a rearrangement/Alder-ene cyclization to afford the new proline framework with a high diastereoselectivity.

## 1. Introduction

**Figure 1:**
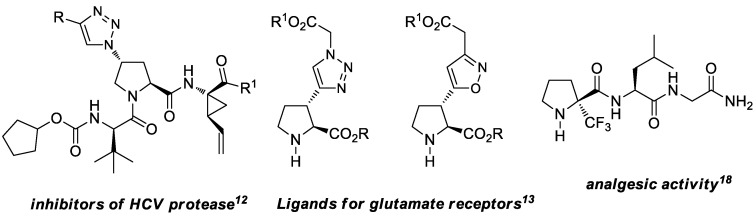
Selected examples of bioactive functionalized prolines.

Proline and its functionalized derivatives are constituents of numerous natural products [1,2,3] and are widely used as pharmaceuticals, in biomedical research and as templates in structure–function relationship studies directed toward the elucidation of biologically active conformations [4,5]. In this context, ring-substituted and quaternary proline analogues are of particular interest [6,7,8,9,10,11,12,13]. Some representative examples of bioactive prolines are depicted in Figure 1. Because of the unique properties of fluorine-containing compounds [14,15,16,17,18], fluorinated α-amino acids, especially their α-fluoromethyl substituted counterparts [19,20,21,22,23], which can function as selective enzyme inhibitors [24,25], are very attractive target molecules for the design of biologically active compounds.

**Scheme 1:**
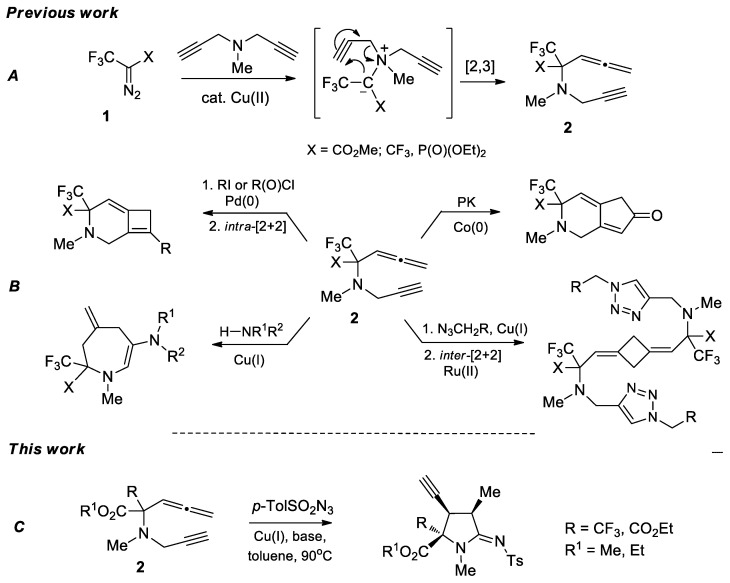
Previous (**A**,**B**) and present (**C**) work.

Recently, we elaborated on a straightforward way to access functionalized allenynes **2** based on the [2,3]-sigmatropic rearrangement of propargyl-containing nitrogen ylides generated in situ from α-CF_3_-diazo Compounds **1** and *N,N*-bis(propargyl)methylamine (Scheme 1A) [26]. Allenynes **2** have proved to be unique doubly unsaturated synthons that can afford a variety of the corresponding α-amino acid derivatives. Thus, the potential of **2** has been clearly revealed in their synthetic transformation under transition metal catalysis, e.g., during Pd-catalyzed Sonogashira coupling followed by intramolecular [2 + 2] cycloaddition [27] and a co-mediated Pauson–Khand reaction [26], Cu-catalyzed tandem amination/cyclization [28] and in intermolecular Ru-catalyzed dimerization [29] (Scheme 1B). Now we wish to disclose a highly diastereoselective pathway to new densely functionalized proline derivatives via a cascade reaction between allenynes **2** and tosylazide, which is involved in Cu(I)-catalyzed alkyne-azide [3 + 2]-cycloaddition, ketenimine rearrangement and Alder-ene cyclization (Scheme 1C). To the best of our knowledge, this type of 1,6-allenyne transformation under metal catalysis to access proline derivatives has been not reported before.

## 2. Results and Discussion

**Scheme 2:**
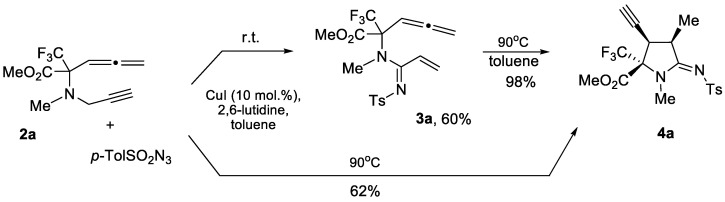
Synthesis of 3-ethynyl-pyrrolidine-2-carboxylate **4a**.

**Figure 2:**
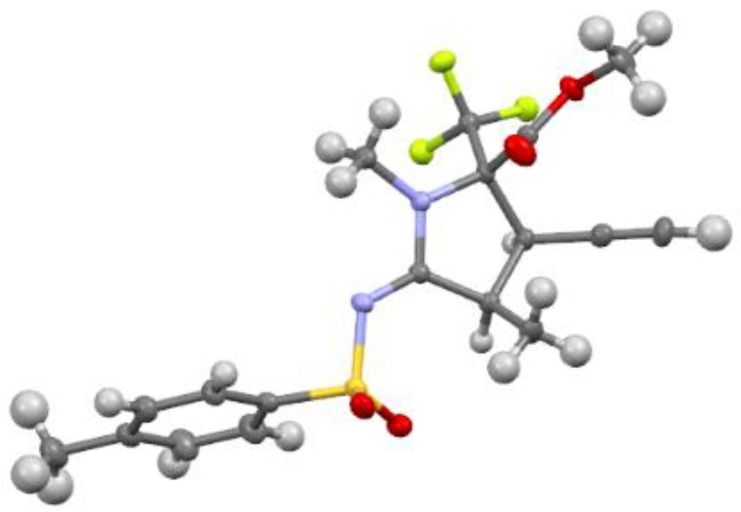
Structure of proline **4a** (CCDC 2208592).

In continuation of our long-term program on the synthesis of new fluorinated amino acids using the transition-metal catalyzed transformation of the unsaturated precursors [30,31,32,33,34], we tested the Cu(I)-catalyzed [3 + 2] cycloaddition reaction of allenyne **2a** with tosylazide, and we initially planned to obtain the corresponding allene-containing 1,2,3-triazole for the investigation of its further chemical behavior, keeping in mind that 1,2,3-triazoles with an electron-withdrawing aryl sulfonyl group on nitrogen are able to form extremely reactive carbenoid species under metal catalysis [35,36,37,38,39]. However, during the course of screening the optimal conditions and catalytic systems, the formation of CF_3_-substituted proline derivative **4a** was unexpectedly revealed in a good yield and had an excellent diastereoselectivity (Scheme 2). The only diastereomer **4a** was easily isolated in its pure form via column chromatography on silica gel; its structure was unambiguously confirmed by NMR-spectroscopy (^1^H, ^13^C and ^19^F) and X-ray analysis (Figure 2). The best yield of proline **4a** (62%) can be achieved by heating equimolar amounts of reagents in toluene at 90 °C in the presence of CuI (10 mol%) and 2,6-dimethylpyridine (1.5 equiv.) as a base for 8 h. The usage of other copper catalysts (CuBr, CuTC, cationic complex Cu(MeCN)_4_PF_6_), organic bases (Et_3_N, DIPEA and pyridines) and solvents (DCE, chloroform and dioxane)) leads to a significant decrease in the product yield.

Then, we discovered that if the reaction is carried out at room temperature, another product, acrylamidine **3a**, is formed. The latter compound was isolated in a 60% yield and was fully characterized using standard physico-chemical methods. It was also found that allene-containing acrylamidine **3a** is able to undergo intramolecular cyclization under heating in toluene at 90 °C in the absence of any catalysts yielding proline **4a** almost quantitatively (Scheme 2).

**Scheme 3:**
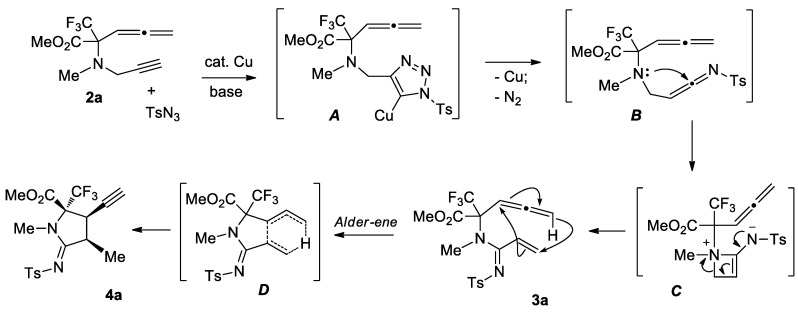
Plausible reaction pathways.

A feasible reaction pathway (Scheme 3) may include the initial formation of copper triazolide **A**, which can be further transformed into the corresponding ketenimine **B** via the release of nitrogen gas [40,41,42,43]. The latter undergoes a skeleton rearrangement via the formation of a relatively unstable four-membered ring intermediate **C,** leading to acrylamidine **3a**. A similar rearrangement has been previously described for the intramolecular annulation of *N*-tethered *N*-sulfonyl-1,2,3-triazoles [44]. Finally, the intramolecular Alder-ene cycloisomerization of allene-containing acrylamidine **3a** (1,6-allenene) occurs with the participation of an allene hydrogen through the concerted six-center transition state **D** to give the product **4a** with a high degree of diastereoselectivity. Such a thermal ene-type reaction, in which the terminal allene acts as the ene-component and the alkene as the enophile, has not been previously described. The closest literature analogy includes the Alder-ene cycloisomerization of 1,6-allenynes, namely α-allenyl propiolamides [45].

**Scheme 4:**
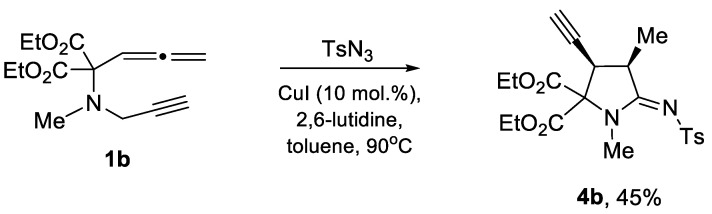
Synthesis of 3-ethynyl-pyrrolidine-2,2-dicarboxylate **4b**.

It turns out that the analogous cascade process also takes place in the case of readily available allenyne **1b [29]** to afford the corresponding proline **4b** as a single diastereomer in an acceptable isolated yield under the same catalytic conditions (Scheme 4).

**Scheme 5:**
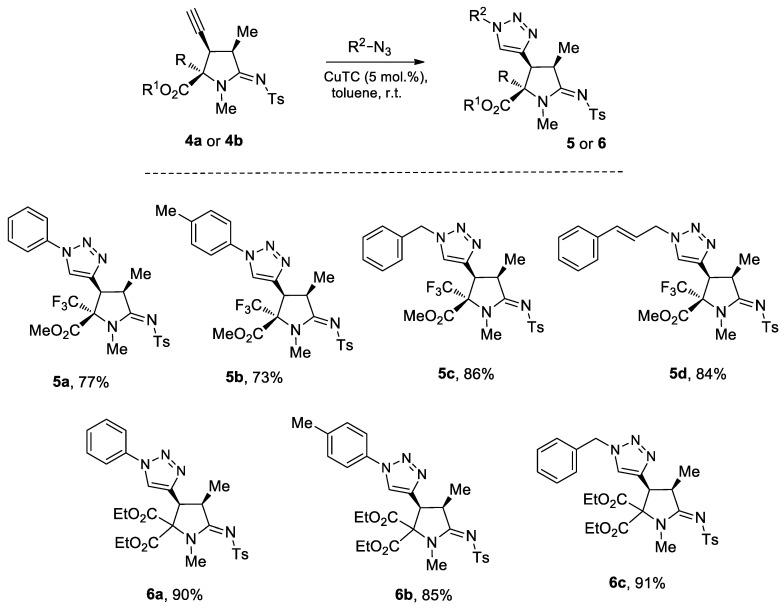
Synthesis of triazoloprolines **5** and **6**.

The presence of an ethynyl group in the structure of prolines **4a,b** makes them unique synthons for further useful transformations. For instance, 3-ethynyl substituted prolines and their triazole-containing derivatives have been recently applied as universal building blocks for the development of new ligands for the activation of ionotropic glutamate receptors, which are important excitatory neurotransmitters in the central nervous system [13,46]. Therefore, in order to demonstrate one of the possible synthetic utilizations of the new compounds **4a,b**, we investigated their Cu(I)-catalyzed alkyne-azide coupling, i.e., the so-called “click” reaction, with alkyl and aryl azides. For these purposes, a series of copper (I)/organic base systems were tested to activate the reaction. As a result, it was revealed that the optimum condition providing the best yields of the target triazoloprolines **5** and **6** was the usage of copper thiophene-2-carboxylate (CuTC) in amounts of 5 mol.% in the absence of any base. The reaction went to completion at room temperature in toluene for 4 h (Scheme 5).

## 3. Materials and Methods

### 3.1. General Information

All the solvents used in the reactions were freshly distilled from appropriate drying agents before use. All the reagents were used as purchased from Sigma-Aldrich (Munich, Germany). An analytical TLC was performed with Merck silica gel 60 F_254_ plates (Darmstadt, Germany), and visualization was accomplished with UV light, iodine vapors or by spraying with Ce(SO_4_)_2_ solution in 5% H_2_SO_4_. Chromatography was carried out using Merck silica gel (Kieselgel 60, 0.063–0.200 mm, Darmstadt, Germany) and petroleum ether/ethyl acetate as an eluent. NMR spectra were obtained with Bruker AV-300 (^1^H, ^19^F) and AV-400 (^1^H, ^13^C, ^19^F) spectrometers (Karlsruhe, Germany), operating at 400 MHz for ^1^H (TMS reference), at 101 MHz for ^13^C, 282 and at 376 MHz for ^19^F (CCl_3_F reference). High-Resolution Mass Spectrometry spectra were carried out using AB Sciex Triple TOF 5600+ (Framingham, MA, USA), which supported different ionization sources. The starting allenynes were synthesized via the previously described protocol. The melting points were determined on a Melting Point Apparatus Stuart SMP 10 (Wertheim, Germany) and are uncorrected.

### 3.2. General Procedure for Preparation of ***4a*** and ***4b***

An oven-dried 10 mL Schlenk tube equipped with a magnetic stirrer was under vacuum and then back-filled with argon. Under a stream of argon, the allenyne (100 mg, 0.404 mmol) in anhydrous toluene (2 mL) was added, followed by the tosyl azide (84 mg, 0.424 mmol, 1.05 equiv.), CuI (7.7 mg, 10 mol.%) and 2,6-luthidine (65 mg, 0.606 mmol, 1.5 equiv.) sequentially. After the reaction mixture was stirred at RT for 4 h and 90 °C overnight, it was cooled to room temperature and concentrated under reduced pressure. The residue was purified by column chromatography on silica gel (petroleum ether/ethyl acetate) to obtain the desired product **4a** and **4b**. This procedure worked perfectly on a 0.5 g scale without decreasing the product yield.

### 3.3. General Procedure for Preparation of ***5a***–***5d*** and ***6a***–***6c***

To a solution of **4a** or **4b** (0.24 mmol) in anhydrous toluene (2 mL), the corresponding amount of azide (0.48 mmol, 2 equiv.) and CuTC (copper (I) thiophene-2-carboxylate) (2.3 mg, 5 mol.%) was added. The reaction mixture was stirred at room temperature for 4 h. Upon the completion of the reaction (monitored) by TLC, the mixed solvent was removed under reduced pressure and the residue was purified by column chromatography on silica gel (petroleum ether/ethyl acetate, ethyl acetate) to obtain the corresponding triazole.

*Methyl 2-(N-methyl-N′-tosylacrylimidamido)-2-(trifluoromethyl)penta-3,4-dienoate (* **3a** *)*


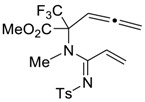


An oven-dried 10 mL Schlenk tube equipped with a magnetic stirrer was under vacuum and then back-filled with argon. Under a stream of argon, the allenyne (100 mg, 0.404 mmol) was added, followed by the tosyl azide (84 mg, 0.424 mmol), CuI (7.7 mg, 10 mol.%) and 2,6-luthidine (65 mg, 0.606 mmol, 1.5 equiv.) in anhydrous toluene (2 mL). After the reaction mixture was stirred at RT for 4h, it was concentrated under reduced pressure. The residue was purified via column chromatography on silica gel (petroleum ether/ethyl acetate) to obtain the desired product **3a**.

Yield: 60% (101 mg) as a light-yellow oil. ^1^H NMR (400 MHz, CDCl_3_) δ 7.72 (d, *J* = 8.2 Hz, 2H), 7.27 (d, *J* = 8.1 Hz, 2H), 6.64 (dd, *J* = 18.0, 12.0 Hz, 1H), 5.83 (d, *J* = 12.0 Hz, 1H), 5.72 (d, *J* = 18.0 Hz, 1H), 5.58 (t, *J* = 6.7 Hz, 1H), 5.05–4.96 (m, 2H), 3.56 (s, 3H), 3.20 (s, 3H), 2.41 (s, 3H). ^19^F NMR (376 MHz, CDCl_3_) δ-67.69. ^13^C NMR (101 MHz, CDCl_3_) δ 208.5, 164.7, 164.1, 142.7, 139.5, 129.1, 128.6, 126.8, 126.2, 123.2 (q, *J* = 288.3 Hz), 85.6, 80.5, 70.5 (q, *J* = 26.8 Hz), 53.3, 36.6, 21.6. HRMS (ESI): calcd. for C_18_H_20_F_3_N_2_O_4_S [M + H]^+^: 417.1090; found: 417.1096.

(2R*,3R*,4R*,Z)-Methyl 3-ethynyl-1,4-dimethyl-5-(tosylimino)-2-(trifluoromethyl)pyrrolidine-2-carboxylate ( **4a** *)*


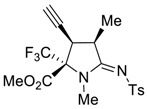


Yield: 62% (104 mg) as a white solid. ^1^H NMR (400 MHz, CDCl_3_) δ 7.81 (d, *J* = 8.2 Hz, 2H), 7.25 (d, *J* = 8.0 Hz, 2H), 3.99 (p, *J* = 7.5 Hz, 1H), 3.87 (s, 3H), 3.64 (dd, *J* = 8.9, 2.5 Hz, 1H), 2.90 (s, 3H), 2.44 (d, *J* = 2.5 Hz, 1H), 2.38 (s, 3H), 1.54 (d, *J* = 7.3 Hz, 1H). ^19^F NMR (376 MHz, CDCl_3_) δ-71.12. ^13^C NMR (101 MHz, CDCl_3_) δ 171.9, 164.8, 142.8, 140.0, 129.4, 126.5, 123.2 (q, *J* = 286.2 Hz), 75.6 (q, *J* = 28.6 Hz), 75.3, 53.8, 38.2, 36.4, 30.9, 21.6, 15.6. HRMS (ESI): calcd. for C_18_H_20_F_3_N_2_O_4_S [M + H]^+^: 417.1090; found: 417.1095.

(3R*,4R*,Z)-Diethyl 3-ethynyl-1,4-dimethyl-5-(tosylimino)pyrrolidine-2,2-dicarboxylate ( **4b** *)*


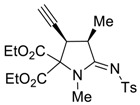


Yield: 45% (79 mg) as a light-yellow oil. ^1^H NMR (400 MHz, CDCl_3_) δ 7.83 (d, *J* = 8.1 Hz, 2H), 7.27 (d, *J* = 7.8 Hz, 2H), 4.37 – 4.28 (m, 4H), 4.09 (dd, *J* = 8.5, 2.3 Hz, 1H), 3.97–3.89 (m, 1H), 3.00 (s, 3H), 2.40 (s, 3H), 2.37 (d, *J* = 2.3 Hz, 1H), 1.52 (d, *J* = 7.4 Hz, 3H), 1.32 (td, *J* = 7.1, 3.9 Hz, 6H). ^13^C NMR (101 MHz, CDCl_3_) δ 172.0, 166.6, 166.0, 142.5, 140.5, 129.3, 126.5, 76.7, 76.2, 76.0, 63.5, 63.0, 38.7, 38.1, 31.9, 21.6, 15.4, 14.2, 14.1. EA calcd. for C_21_H_26_N_2_O_6_S (%): C, 58.05; H, 6.03; N, 6.45. Found: C, 57.93; H, 5.99; N, 6.40.

(2R*,3S*,4R*,Z)-Methyl 1,4-dimethyl-3-(1-phenyl-1H-1,2,3-triazol-4-yl)-5-(tosylimino)-2-(trifluoromethyl)pyrrolidine-2-carboxylate ( **5a** *)*


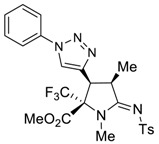


Yield: 77% (99 mg) as a white solid. M.p. 147–149 °C. ^1^H NMR (400 MHz, CDCl_3_) δ 7.90 (s, 1H), 7.87 (d, *J* = 8.0 Hz, 2H), 7.73 (d, *J* = 7.1 Hz, 2H), 7.55 (t, *J* = 7.7 Hz, 2H), 7.47 (t, *J* = 7.8 Hz, 1H), 7.30 (d, *J* = 8.0 Hz, 2H), 4.26 (d, *J* = 8.8 Hz, 1H), 4.20–4.13 (m, 1H), 3.86 (s, 3H), 3.02 (s, 3H), 2.43 (s, 3H), 1.49 (d, *J* = 7.4 Hz, 3H). ^13^C NMR (101 MHz, CDCl_3_) δ 173.0, 165.3, 142.7, 140.4, 140.3, 136.7, 129.9, 129.4, 129.1, 126.5, 123.8 (q, *J* = 286.6 Hz), 122.3, 120.4, 75.6 (q, *J* = 28.3 Hz), 54.0, 40.6, 40.2, 30.8, 21.6, 16.1. ^19^F NMR (376 MHz, CDCl_3_) δ-70.51. EA calcd. for C_24_H_24_F_3_N_5_O_4_S (%): C, 53.83; H, 4.52; N, 13.08. Found: C, 54.08; H, 4.55; N. 13.17.

(2R*,3S*,4R*,Z)-Methyl 1,4-dimethyl-3-(1-p-tolyl-1H-1,2,3-triazol-4-yl)-5-(tosylimino)-2-(trifluoromethyl)pyrrolidine-2-carboxylate ( **5b** *)*


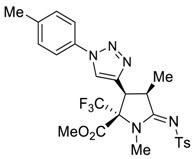


Yield: 73% (96 mg) as a white solid. M.p. 162–164 °C. ^1^H NMR (300 MHz, CDCl_3_) δ 7.91–7.83 (m, 3H), 7.59 (d, *J* = 8.3 Hz, 2H), 7.36–7.27 (m, 4H), 4.26 (d, *J* = 8.9 Hz, 1H), 4.20–4.11 (m, 1H), 3.86 (s, 3H), 3.02 (s, 3H), 2.43 (s, 6H), 1.48 (d, *J* = 7.3 Hz, 3H). ^19^F NMR (376 MHz, CDCl_3_) δ-70.53. ^13^C NMR (101 MHz, CDCl_3_) δ 173.0, 165.3, 142.7, 140.3, 140.2, 139.3, 134.4, 130.4, 129.4, 126.5, 123.8 (q, *J* = 286.8 Hz), 122.2, 120.4, 75.6 (q, *J* = 28.0 Hz), 54.0, 40.6, 40.2, 30.9, 21.6, 21.2, 16.1. EA calcd. for C_25_H_26_F_3_N_5_O_4_S (%): C, 54.64; H, 4.77; N, 12.74. Found: C, 54.62; H, 4.82; N, 12.75.

(2R*,3S*,4R*,Z)-methyl 3-(1-benzyl-1H-1,2,3-triazol-4-yl)-1,4-dimethyl-5-(tosylimino)-2-(trifluoromethyl)pyrrolidine-2-carboxylate ( **5c** *)*


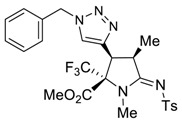


Yield: 86% (113 mg) as a white solid. M.p. 158–160 °C. ^1^H NMR (400 MHz, CDCl_3_) δ 7.85 (d, *J* = 8.2 Hz, 2H), 7.43–7.35 (m, 3H), 7.33 (s, 1H), 7.28 (d, *J* = 8.2 Hz, 2H), 7.22 (d, *J* = 5.3 Hz, 2H), 5.60 (d, *J* = 14.9 Hz, 1H), 5.46 (d, *J* = 14.9 Hz, 1H), 4.17 (d, *J* = 8.9 Hz, 1H), 4.05 (p, *J* = 7.7 Hz, 1H), 3.69 (s, 3H), 2.97 (s, 3H), 2.41 (s, 3H), 1.40 (d, *J* = 7.4 Hz, 3H). ^13^C NMR (101 MHz, CDCl_3_) δ 173.0, 165.4, 142.7, 140.3, 140.1, 134.5, 129.4, 129.1, 128.0, 126.5, 123.8 (q, *J* = 287.0 Hz), 123.8, 75.7 (q, *J* = 28.8 Hz), 54.3, 53.8, 40.8, 40.0, 30.9, 21.7, 16.2. ^19^F NMR (282 MHz, CDCl_3_) δ-70.50. EA calcd. for C_25_H_26_F_3_N_5_O_4_S (%): C, 54.64; H, 4.77; N, 12.74. Found: C, 54.47; H, 4.86; N, 12.73.

(2R*,3S*,4R*,Z)-methyl 3-(1-cinnamyl-1H-1,2,3-triazol-4-yl)-1,4-dimethyl-5-(tosylimino)-2-(trifluoromethyl)pyrrolidine-2-carboxylate ( **5d** *)*


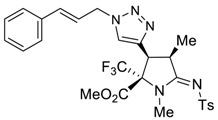


Yield: 84% (116 mg) as a white solid. M.p. 136–138 °C. ^1^H NMR (400 MHz, Chloroform-*d*) δ 7.83 (d, *J* = 8.1 Hz, 2H), 7.51 (s, 1H), 7.37–7.30 (m, 4H), 7.29 (d, *J* = 5.1 Hz, 1H), 7.24 (s, 2H), 6.58 (d, *J* = 15.8 Hz, 1H), 6.36–6.26 (m, 1H), 5.10 (t, *J* = 6.8 Hz, 2H), 4.18 (d, *J* = 8.9 Hz, 1H), 4.09–4.04 (m, 1H), 3.77 (s, 3H), 2.97 (s, 3H), 2.38 (s, 3H), 1.40 (d, *J* = 7.4 Hz, 3H). ^13^C NMR (101 MHz, CDCl_3_) δ 173.0, 165.3, 142.6, 140.3, 139.8, 135.6, 135.4, 129.3, 128.9, 128.7, 126.8, 126.4, 123.9, 123.8 (q, *J* = 286.2 Hz), 121.6, 75.7 (q, *J* = 27.8 Hz), 53.9, 52.4, 40.7, 40.1, 30.8, 21.6, 16.1. ^19^F NMR (376 MHz, CDCl_3_) δ -70.5. EA calcd. for C_27_H_28_F_3_N_5_O_4_S (%): C, 56.34; H, 4.90; N, 12.17. Found: C, 56.21; H, 4.97; N, 12.29.

(3S*,4R*,Z)-diethyl 1,4-dimethyl-3-(1-phenyl-1H-1,2,3-triazol-4-yl)-5-(tosylimino) pyrrolidine-2,2-dicarboxylate ( **6a** *)*


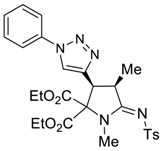


Yield: 90% (120 mg) as a white solid. M.p. 137–139 °C. ^1^H NMR (400 MHz, CDCl_3_) δ 7.95 (s, 1H), 7.84 (d, *J* = 7.9 Hz, 2H), 7.70 (d, *J* = 7.8 Hz, 2H), 7.50 (t, *J* = 7.7 Hz, 2H), 7.41 (t, *J* = 7.4 Hz, 1H), 7.25 (d, *J* = 8.2 Hz, 2H), 4.64 (d, *J* = 8.5 Hz, 1H), 4.25 (q, *J* = 7.1 Hz, 2H), 4.22–4.15 (m, 1H), 4.12–4.04 (m, 2H), 3.09 (s, 3H), 2.38 (s, 3H), 1.50 (d, *J* = 7.3 Hz, 3H), 1.24 (t, *J* = 7.3 Hz, 3H), 1.08 (t, *J* = 7.1 Hz, 3H). ^13^C NMR (101 MHz, CDCl_3_) δ 173.0, 166.8, 166.5, 142.4, 141.5, 140.7, 136.9, 129.9, 129.3, 128.9, 126.4, 122.3, 120.4, 63.2, 62.9, 42.9, 40.1, 32.1, 21.6, 15.9, 14.0, 13.8. EA calcd. for C_27_H_31_N_5_O_6_S (%): C, 58.58; H, 5.64; N, 12.65. Found: C, 58.66; N, 5.67; H, 12.68.

(3S*,4R*,Z)-diethyl 1,4-dimethyl-3-(1-p-tolyl-1H-1,2,3-triazol-4-yl)-5-(tosylimino) pyrrolidine-2,2-dicarboxylate ( **6b** *)*


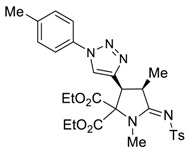


Yield: 85% (116 mg) as a white solid. M.p. 157–158 °C. ^1^H NMR (400 MHz, CDCl_3_) δ 7.92 (s, 1H), 7.86 (d, *J* = 8.0 Hz, 2H), 7.59 (d, *J* = 8.3 Hz, 2H), 7.31 (d, *J* = 8.1 Hz, 2H), 7.29–7.26 (d, *J* = 7.1 Hz, 2H), 4.66 (d, *J* = 8.4 Hz, 1H), 4.27 (q, *J* = 6.9 Hz, 2H), 4.23–4.16 (m, 1H), 4.14–4.04 (m, 2H), 3.10 (s, 3H), 2.41 (s, 3H), 2.40 (s, 3H), 1.52 (d, *J* = 7.4 Hz, 3H), 1.26 (t, *J* = 7.3 Hz, 3H), 1.09 (t, *J* = 7.2 Hz, 3H). ^13^C NMR (101 MHz, CDCl_3_) δ 173.0, 166.8, 166.6, 142.4, 141.4, 140.7, 139.1, 134.6, 130.4, 129.3, 126.4, 122.2, 120.4, 63.2, 62.9, 43.0, 40.2, 32.1, 21.6, 21.2, 15.9, 14.0, 13.8. EA calcd. for C_28_H_33_N_5_O_6_S (%): C, 59.24; H, 5.86; N, 12.34. Found: C, 58.99; H, 5.89; N, 12.38.

(3S*,4R*,Z)-diethyl 3-(1-benzyl-1H-1,2,3-triazol-4-yl)-1,4-dimethyl-5-(tosylimino) pyrrolidine-2,2-dicarboxylate ( **6c** *)*


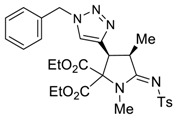


Yield: 91% (124 mg) as a colorless oil. ^1^H NMR (400 MHz, CDCl_3_) δ 7.83 (d, *J* = 8.0 Hz, 2H), 7.39 (s, 1H), 7.37–7.32 (m, 3H), 7.26–7.24 (m, 2H), 7.23–7.19 (m, 2H), 5.54 (d, *J* = 14.9 Hz, 1H), 5.45 (d, *J* = 14.9 Hz, 1H), 4.54 (d, *J* = 8.5 Hz, 1H), 4.21 (q, *J* = 7.1 Hz, 2H), 4.14–4.06 (m, 1H), 4.01–3.87 (m, 2H), 3.05 (s, 3H), 2.39 (s, 3H), 1.41 (d, *J* = 7.4 Hz, 3H), 1.19 (t, *J* = 7.1 Hz, 3H), 1.00 (t, *J* = 7.2 Hz, 3H). ^13^C NMR (101 MHz, CDCl_3_) δ 173.0, 166.8, 166.6, 142.4, 141.3, 140.8, 134.8, 129.3, 129.3, 128.9, 128.0, 126.4, 123.9, 63.1, 62.8, 54.2, 43.1, 40.1, 32.1, 21.6, 15.8, 14.0, 13.7. EA calcd. for C_28_H_33_N_5_O_6_S (%): C, 59.24; H, 5.86; N, 12.34. Found: C, 59.03; H, 5.85; N, 12.13.

### 3.4. X-ray Structure Determination of ***4a***

Crystals (C_18_H_19_F_3_N_2_O_4_S, *M* = 416.41) were monoclinic and had a space group *P*2_1_/c, at 120K *a* = 9.7168(13), *b* = 16.001(2), *c* = 12.2398(17) Å, *β* = 99.355(3)°, *V* = 1877.7(4) Å^3^, *Z* = 4, *d*_calc._ = 1.473 g/cm^3^, *μ* = 2.29 cm^−1^. Data collection was carried out with a Bruker SMART APEX II diffractometer, *λ*(MoKα) = 0.71073 Å, *ω*-scan technique, *T* = 120(2) K, 3670 independent reflections (*R*_int_ = 0.0674) with *2θ*_max_ *=* 52.0° were collected and used in refinement. The structure was solved with direct methods and were refined using the full matrix least-squares technique against *F*^2^ with the anisotropic thermal parameters for all non-hydrogen atoms. At the final stage, the structure was refined as a 2-component twin (the BASF was 0.291(2)). The hydrogen atoms were placed geometrically and were included in the structure factors calculations in the riding motion approximation. The refinement converged to *wR*_2_ = 0.1177 and GOF = 1.027 for all the independent reflections (*R*_1_ = 0.0492 was calculated against *F* for 2834 observed reflections with I > 2σ(I)). All the calculations were performed using the SHELXL program package [47]. CCDC deposition number 2208592 contains the supplementary crystallographic data for this paper. These data can be obtained free of charge from The Cambridge Crystallographic Data Centre.

## 4. Conclusions

In conclusion, we elaborated on an efficient pathway for densely functionalized proline derivatives. The method is based on a Cu(I)-catalyzed reaction between CF_3_-substituted allenynes and tosylazide, which involves the cascade of [3 + 2]-cycloaddition/ketenimine and a rearrangement/Alder-ene cyclization to afford the new 3-ethynyl proline derivatives in moderate-to-good yields and with a high diastereoselectivity. The synthetic potential of the latter compounds was demonstrated in a Cu(I)-catalyzed “click” reaction with alkyl and aryl azides, which provided access to the corresponding triazole-containing prolines as single diastereomers in high yields.

## Data Availability

Data are contained within the article and Supplementary Materials.

## Supplementary Materials

The following supporting information can be downloaded at: https://www.mdpi.com/article/10.3390/molecules27206898/s1. The following are available online: copies of the ^1^H, ^19^F and ^13^C NMR spectra for all novel compounds (Figures S1–S26).

## Sample Availability

Samples of all the compounds are available from the authors.
