# Supplementary material for: Diastereoselective Synthesis of Highly Functionalized Proline Derivatives
Source: Molecules. 2022 Oct 14;27(20):6898. doi: 10.3390/molecules27206898 (PMC9609175; doi:10.3390/molecules27206898)
Supplement: Supplementary file 1 [file molecules-27-06898-s001.zip › molecules-1966288-supplementary.pdf]

NMR spectra

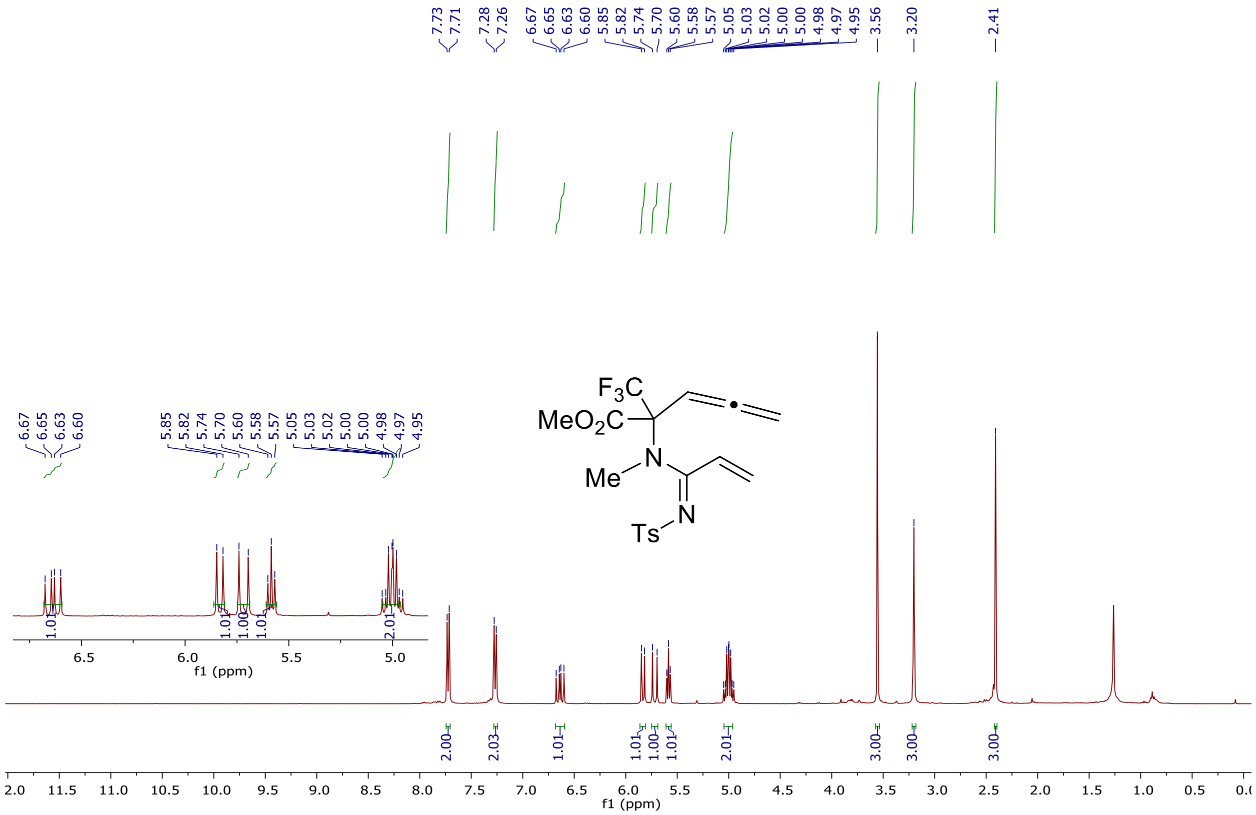

Figure S1. ^{1}H NMR spectrum of **3a** in $CDCl_3$

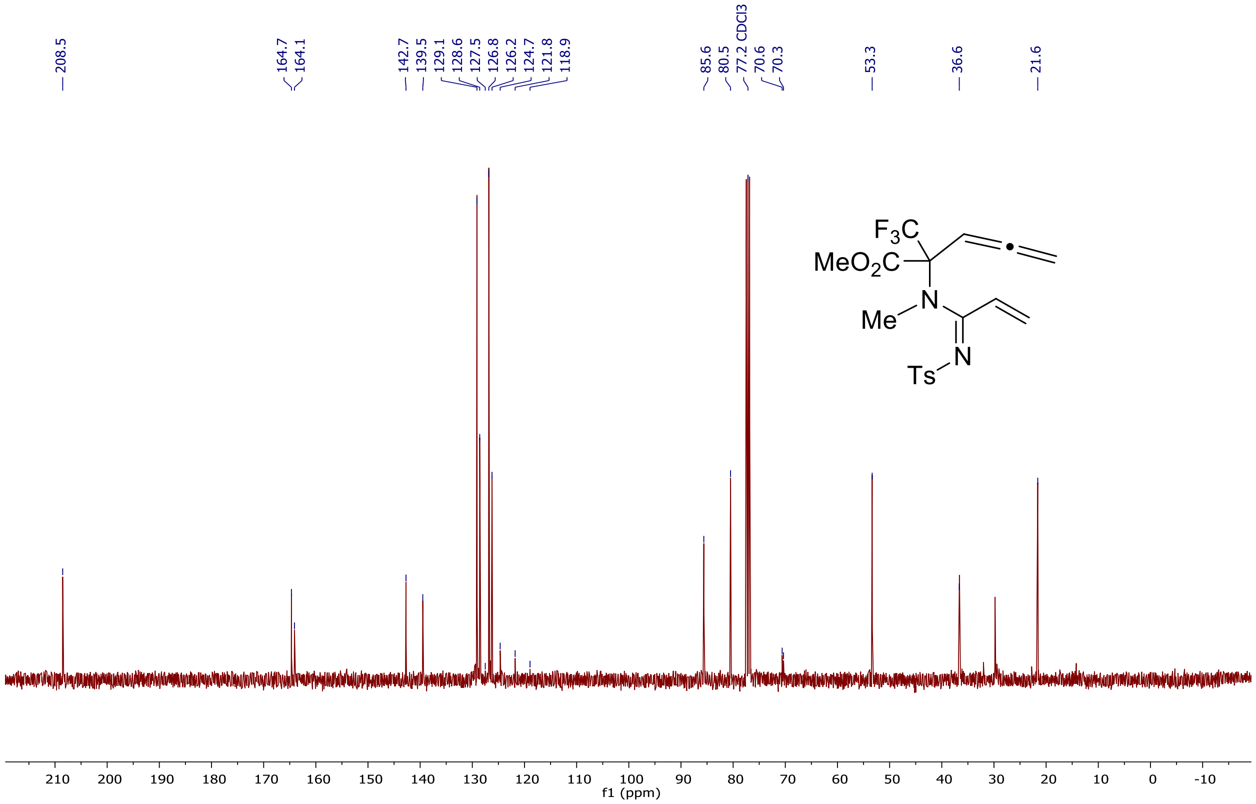

Figure S2. ^{13}C NMR spectrum of **3a** in $CDCl_3$

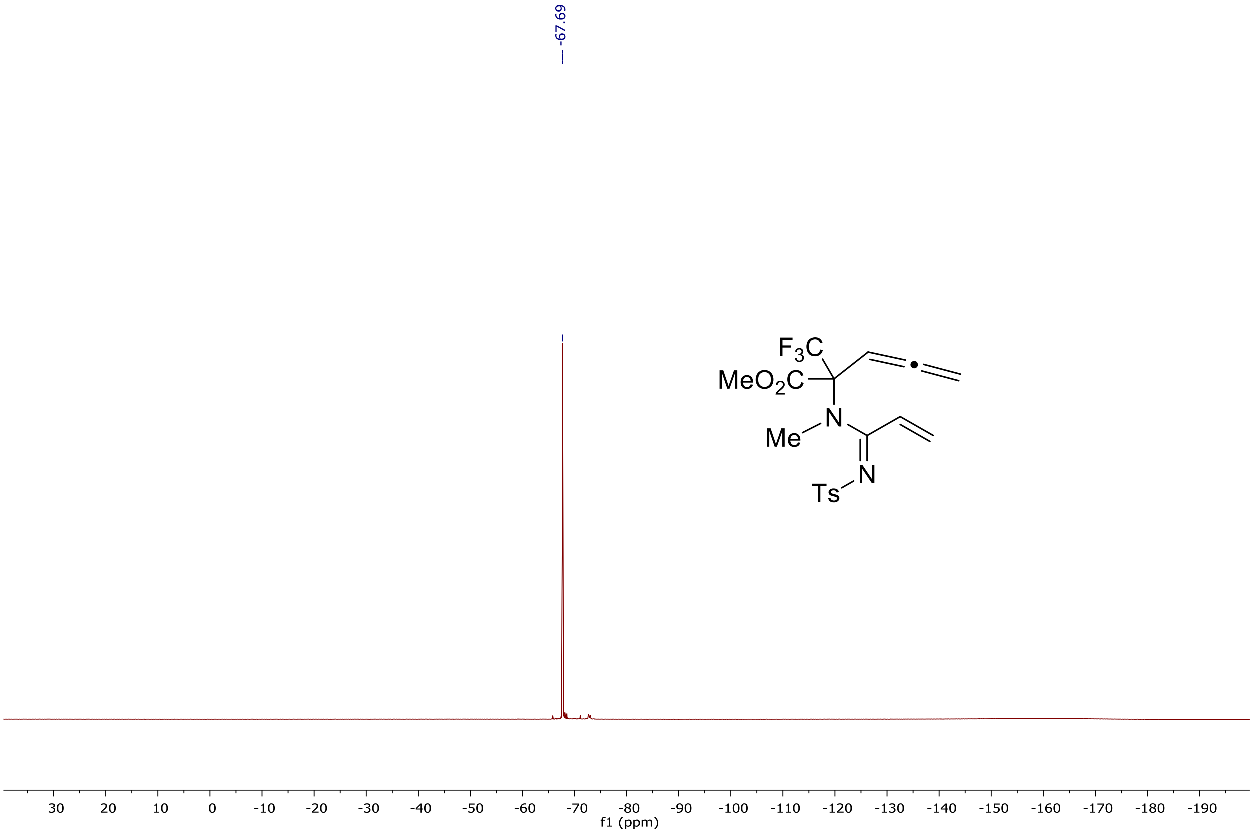

Figure S3. ^{19}F NMR spectrum of **3a** in $CDCl_3$

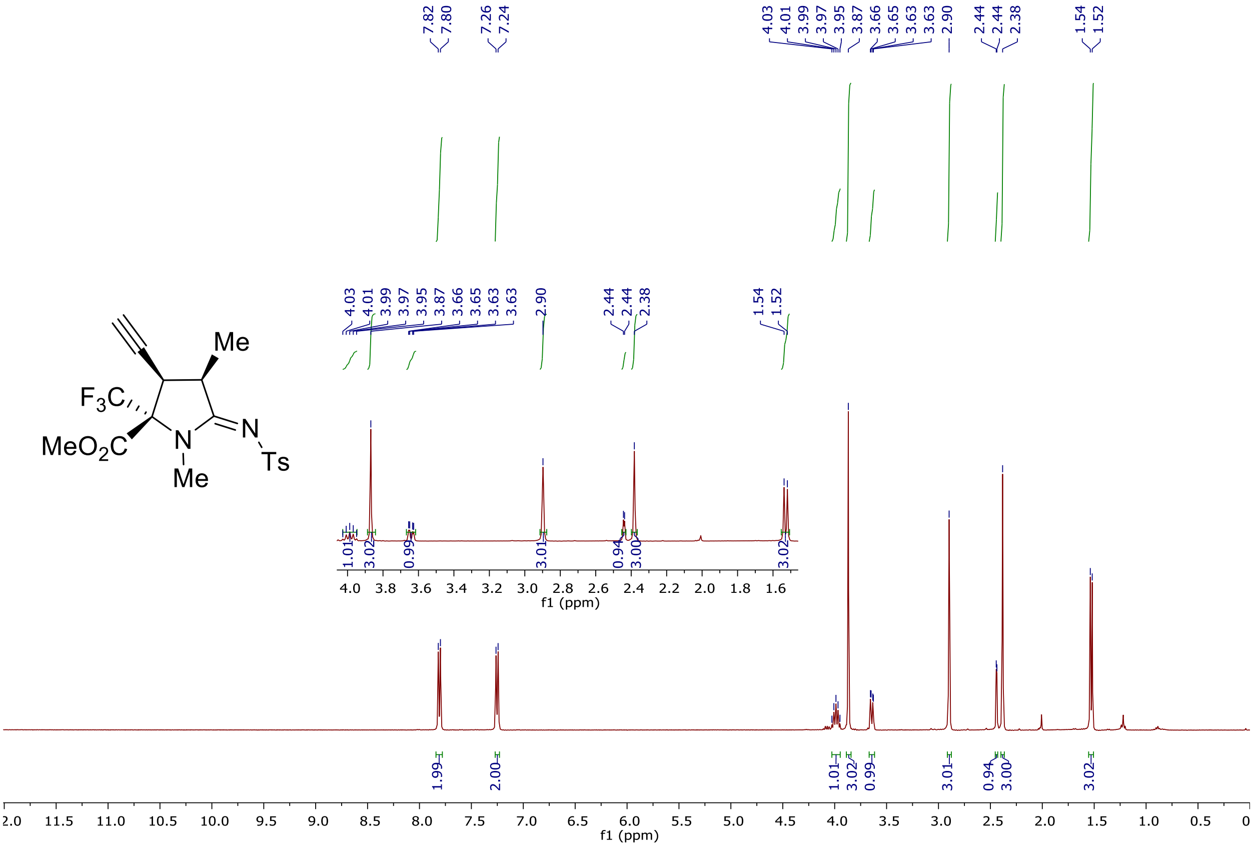

Figure S4. ^{1}H NMR spectrum of **4a** in $CDCl_3$

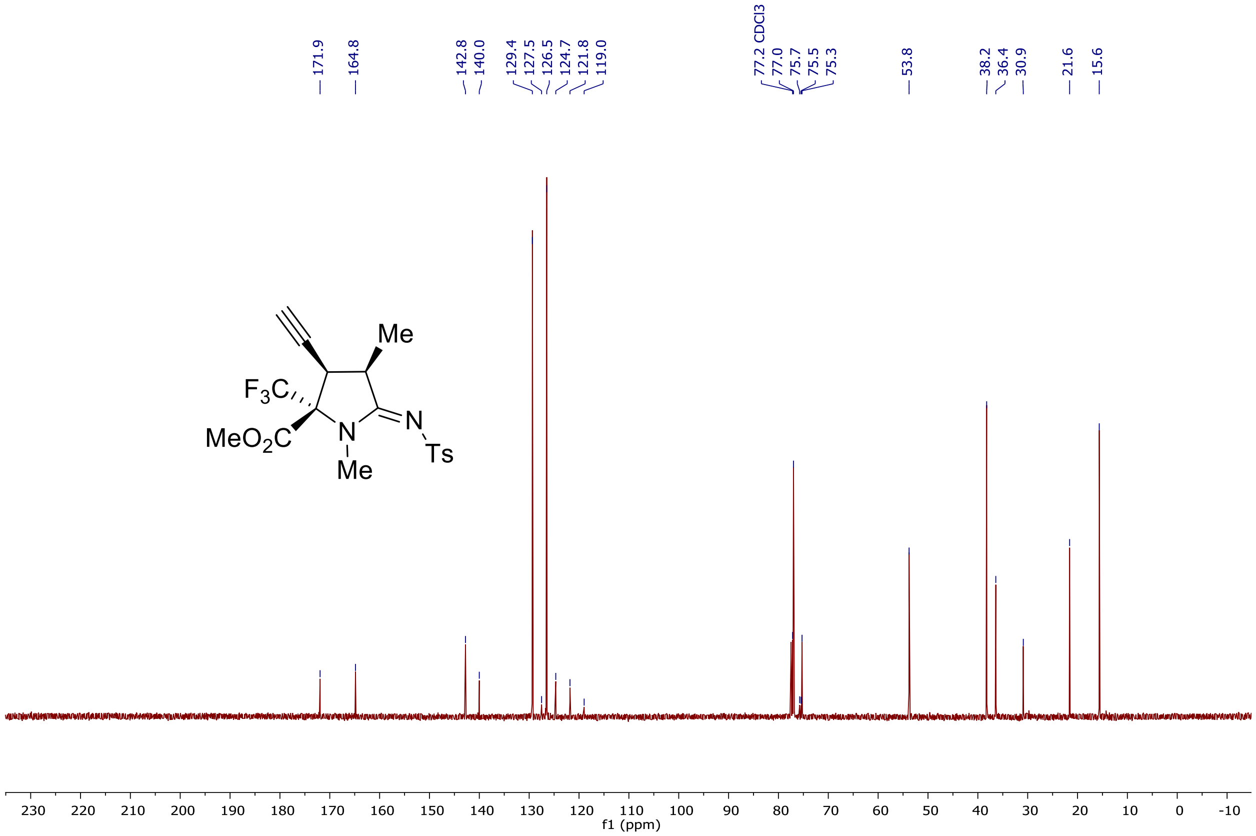

Figure S5. ^{13}C NMR spectrum of **4a** in $CDCl_3$

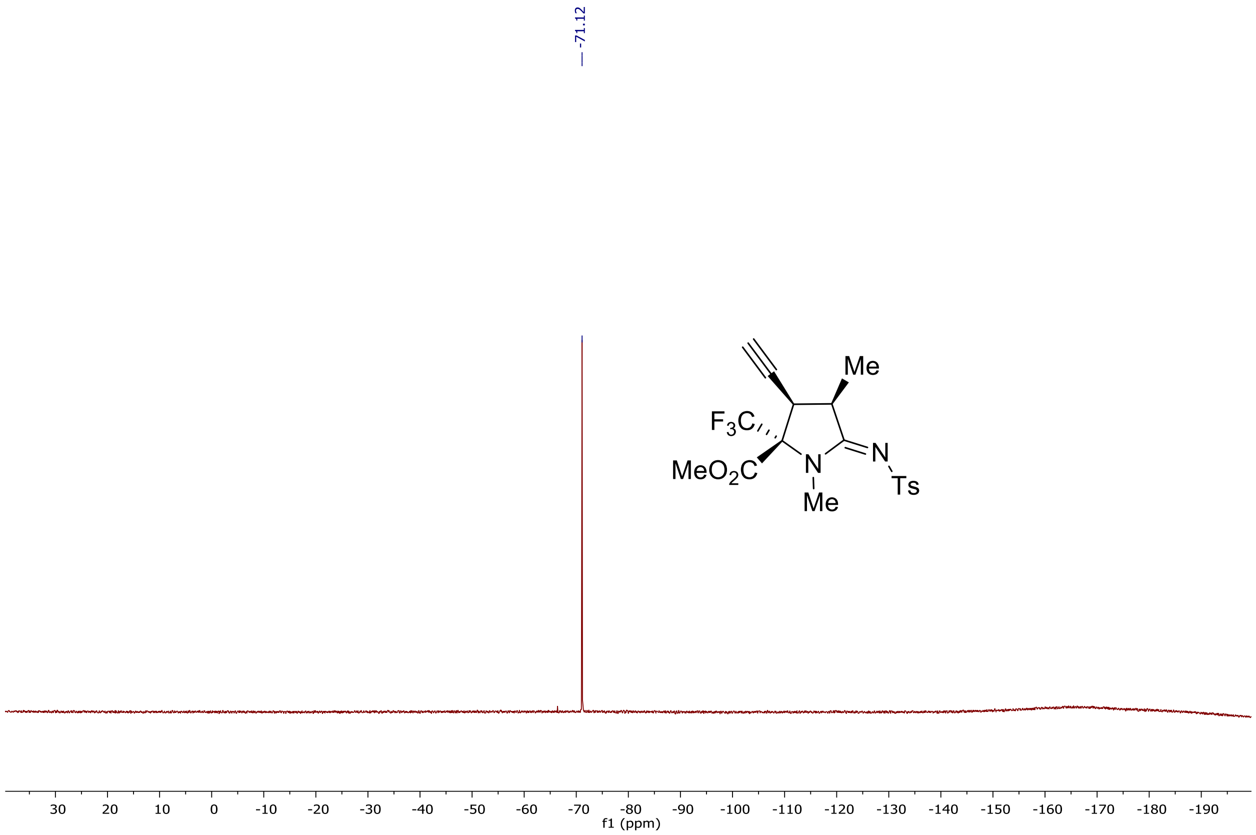

Figure S6. ^{19}F NMR spectrum of **4a** in $CDCl_3$

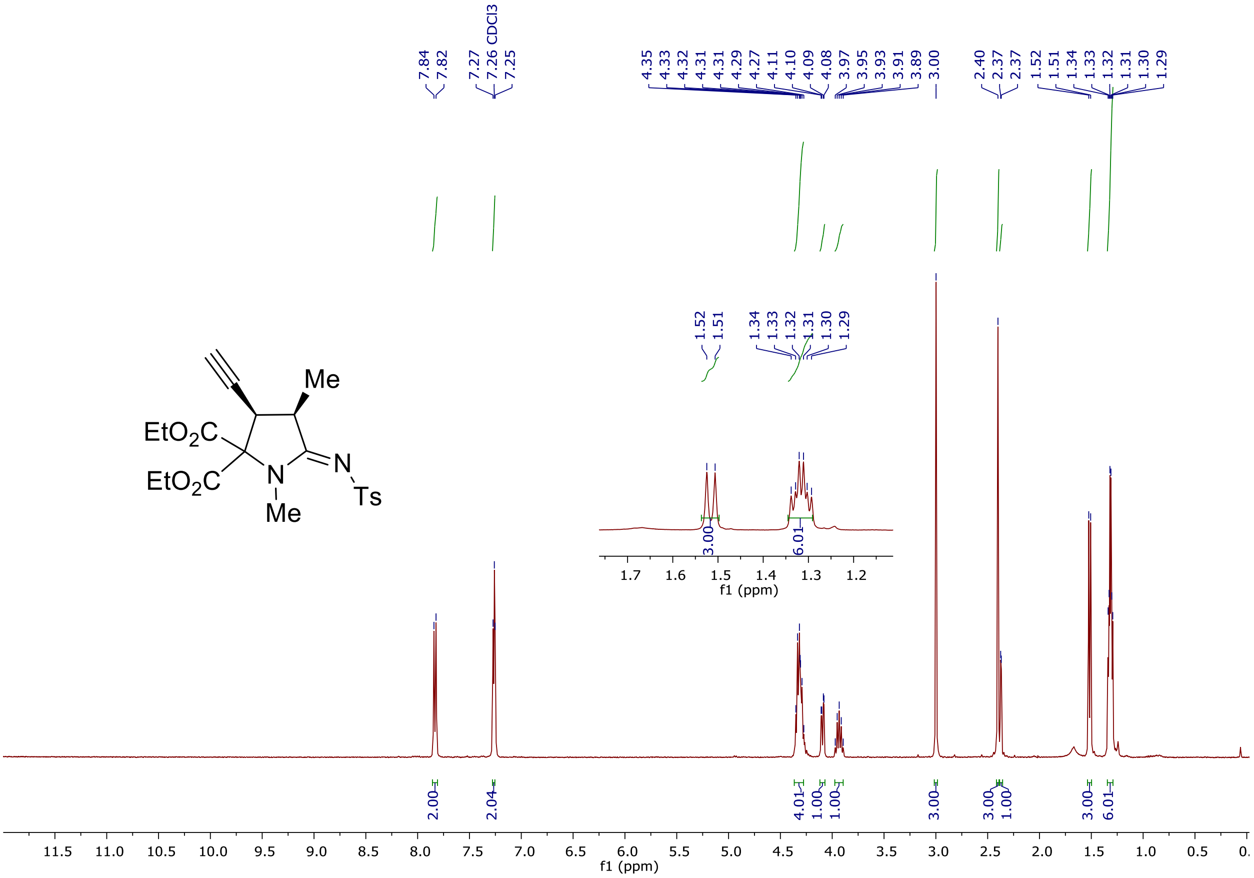

Figure S7. ^{1}H NMR spectrum of **4b** in $CDCl_3$

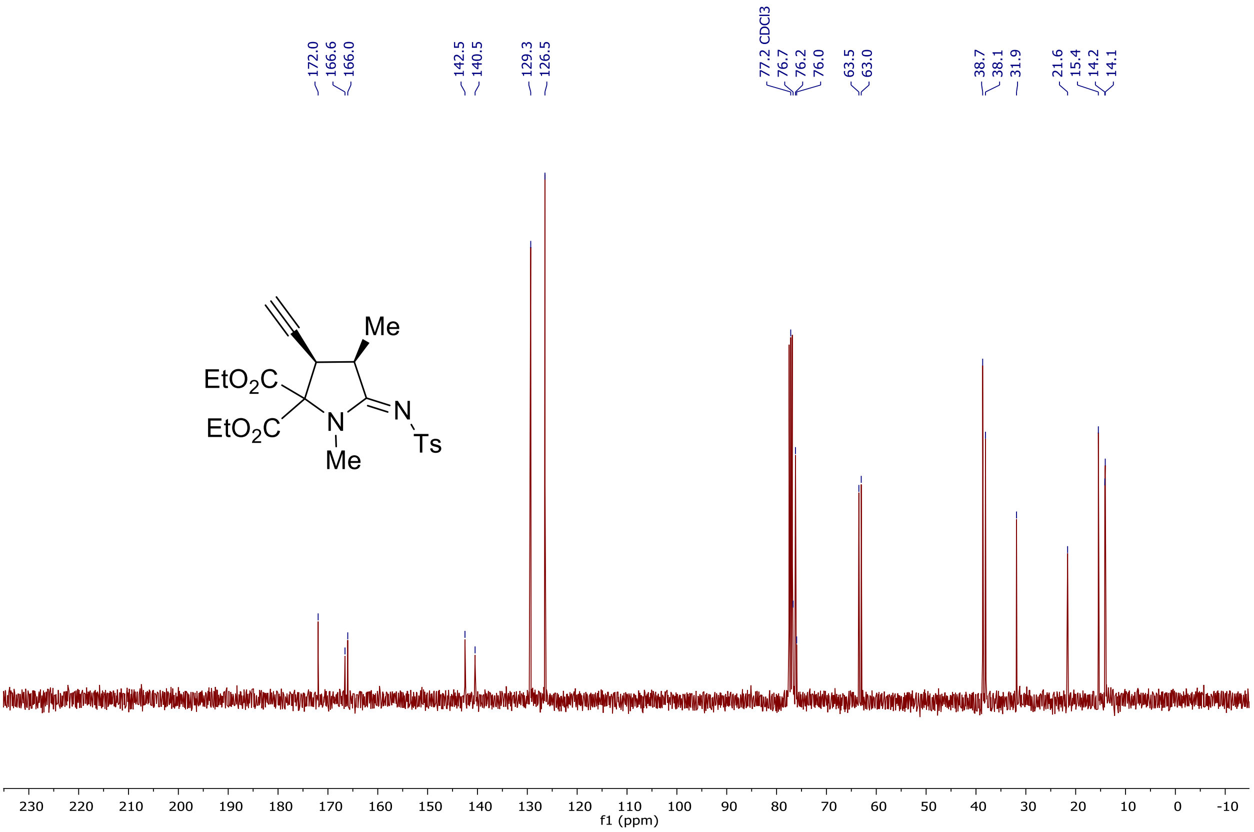

Figure S8. ^{13}C NMR spectrum of **4b** in $CDCl_3$

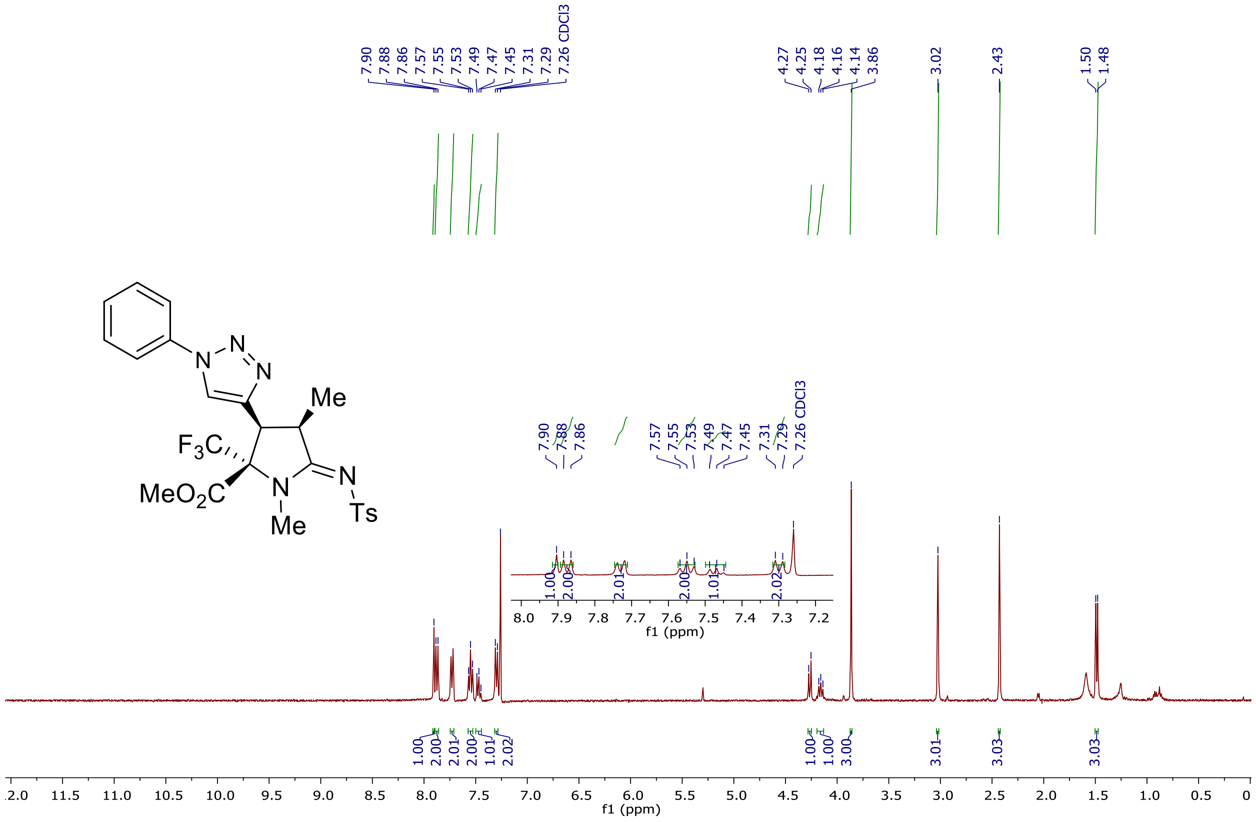

Figure S9. ^{1}H NMR spectrum of **5a** in $CDCl_3$

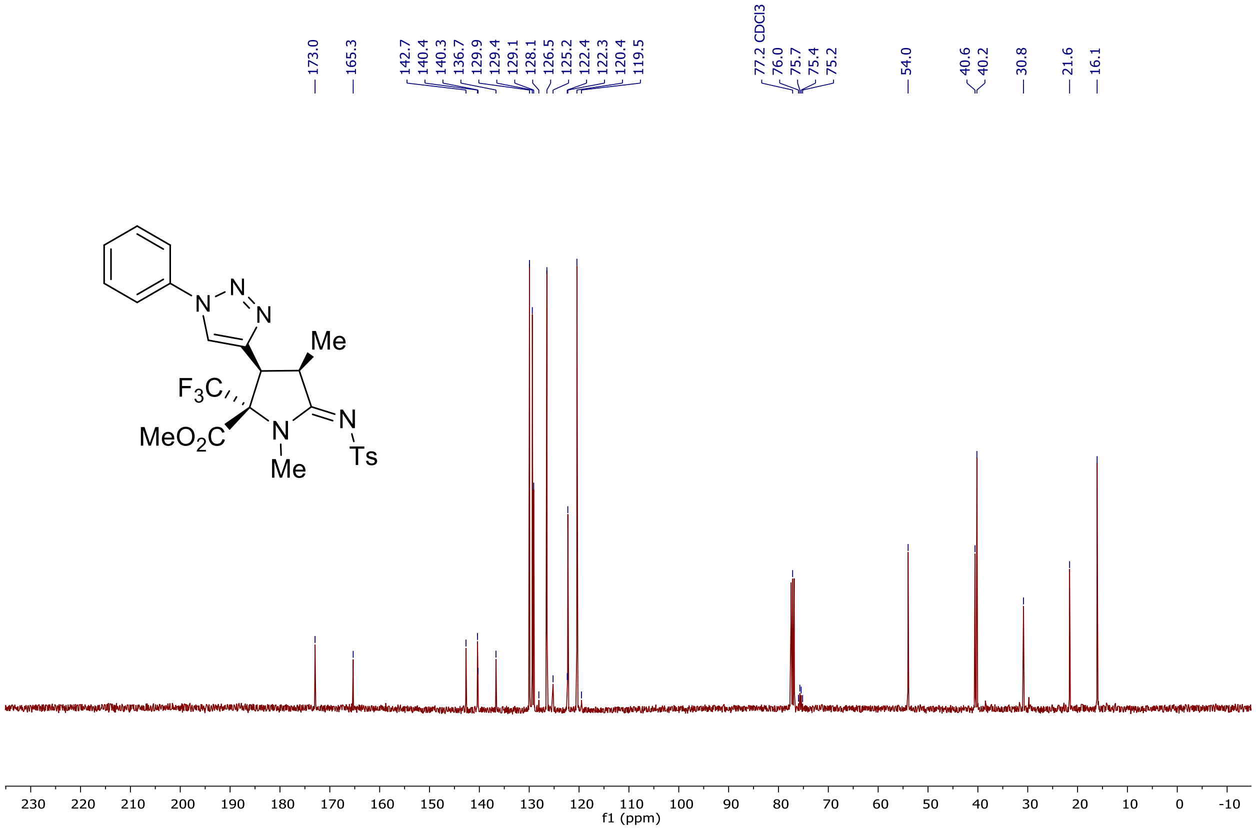

Figure S10. ^{13}C NMR spectrum of **5a** in $CDCl_3$

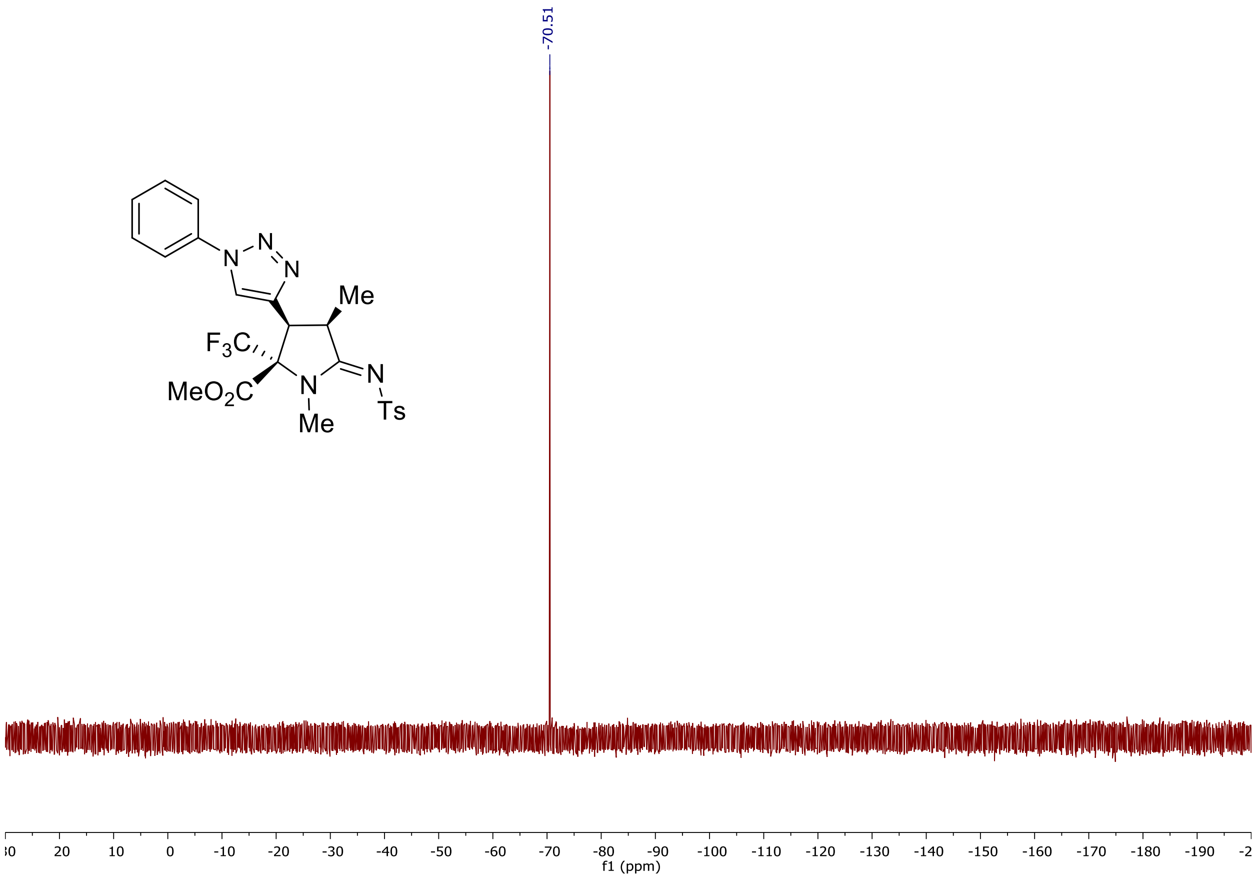

Figure S11. ^{19}F NMR spectrum of **5a** in $CDCl_3$

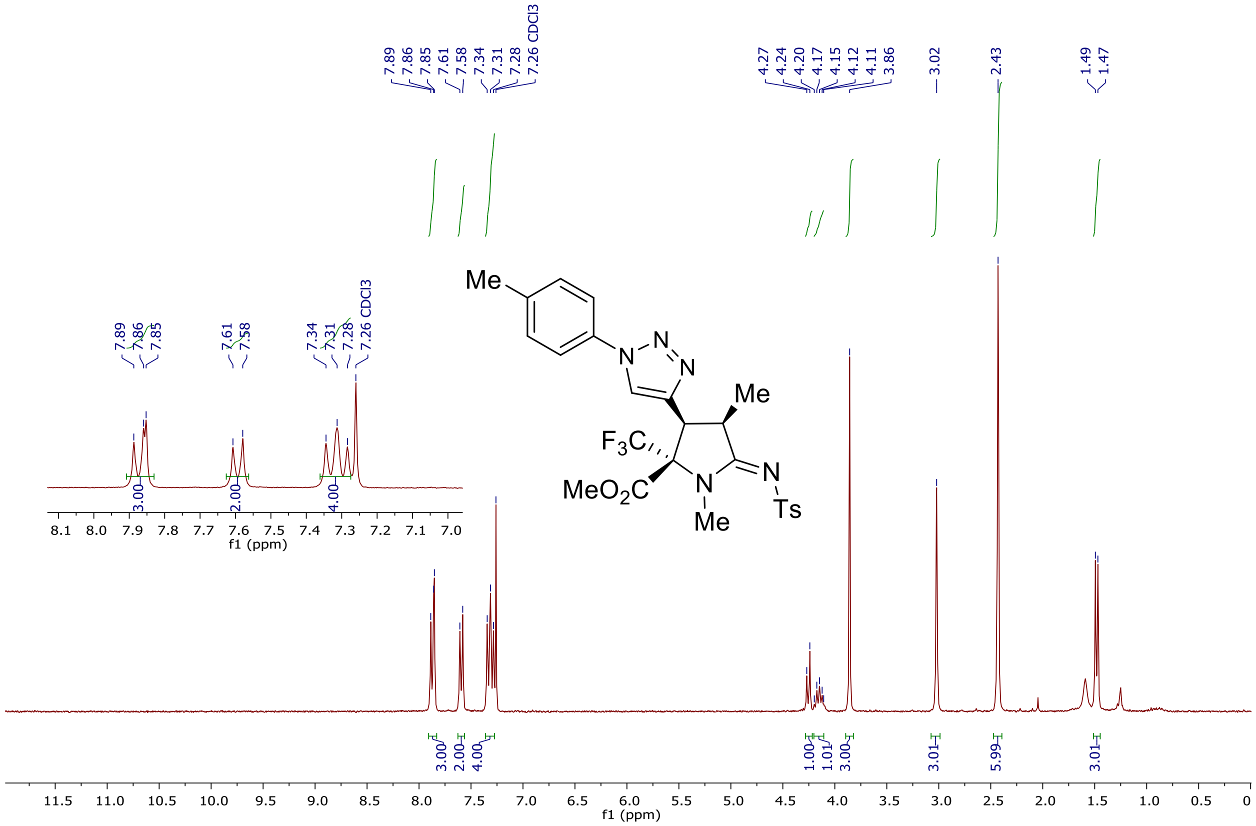

Figure S12. ^{1}H NMR spectrum of **5b** in $CDCl_3$

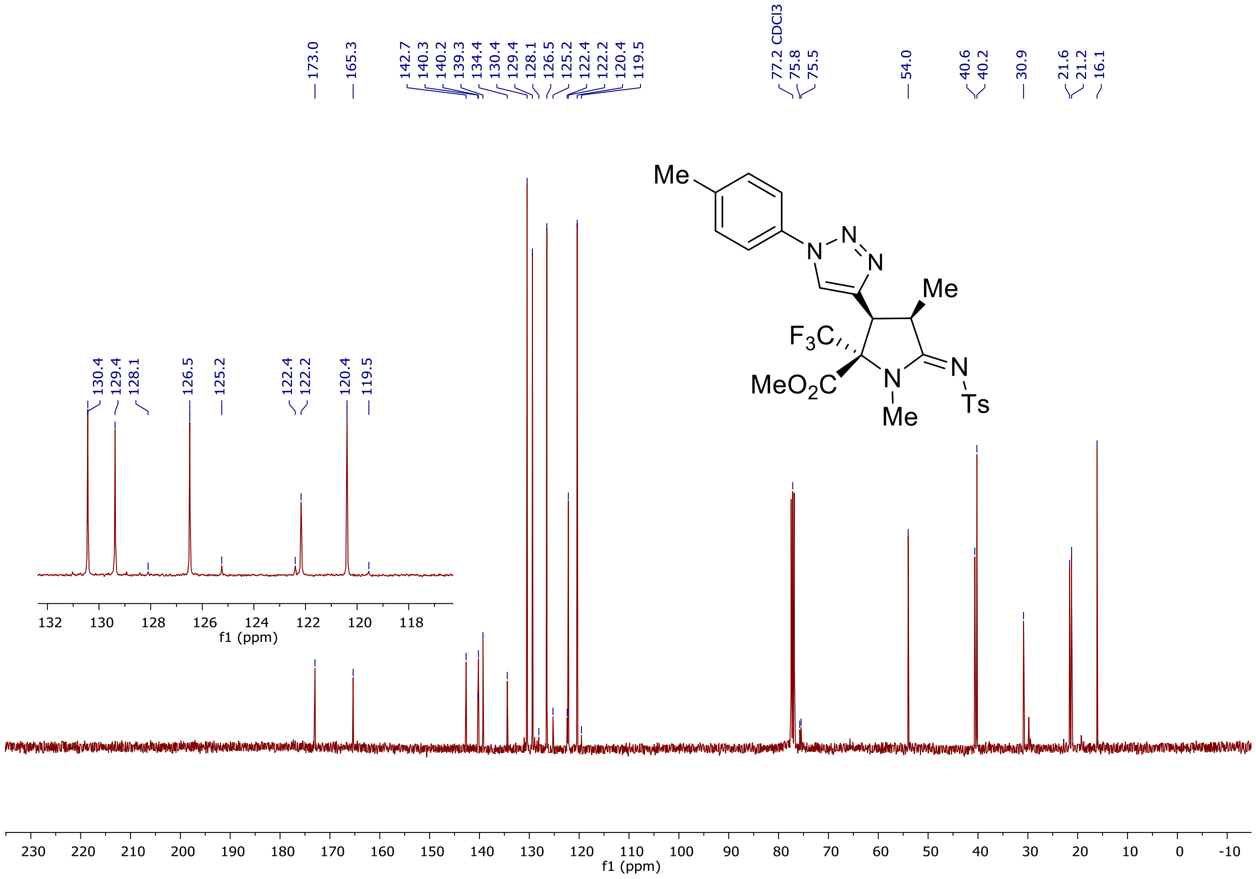

Figure S13. ^{13}C NMR spectrum of **5b** in $CDCl_3$

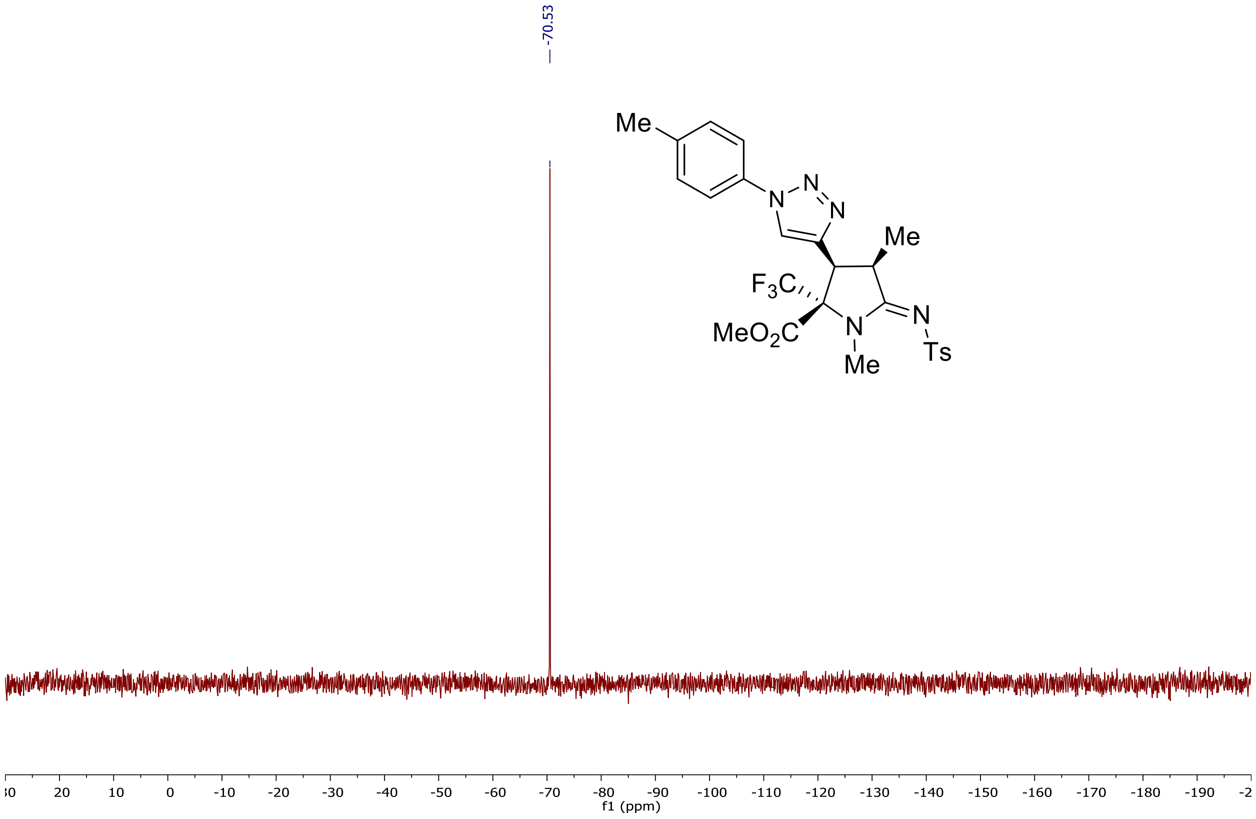

Figure S14. ^{19}F NMR spectrum of **5b** in $CDCl_3$

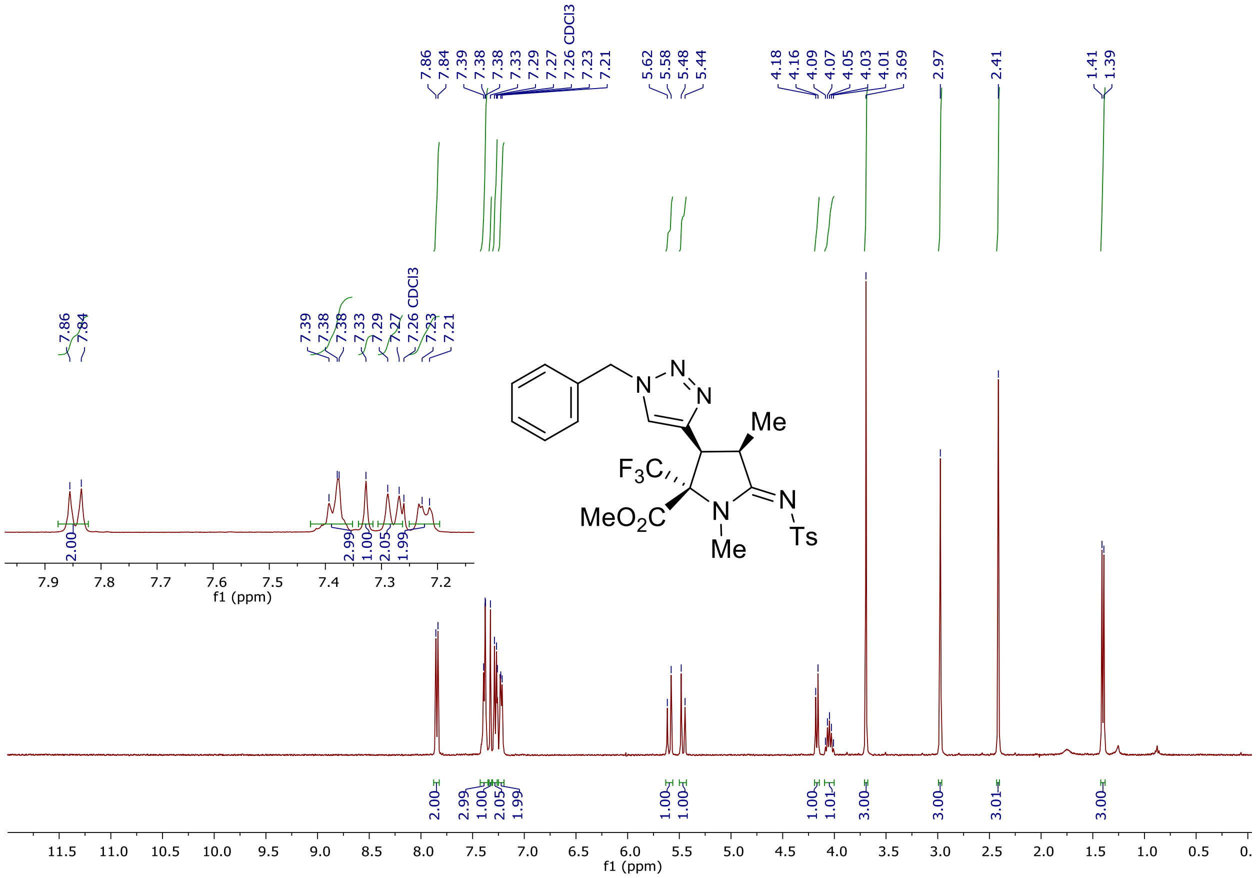

Figure S15. ^{1}H NMR spectrum of **5c** in $CDCl_3$

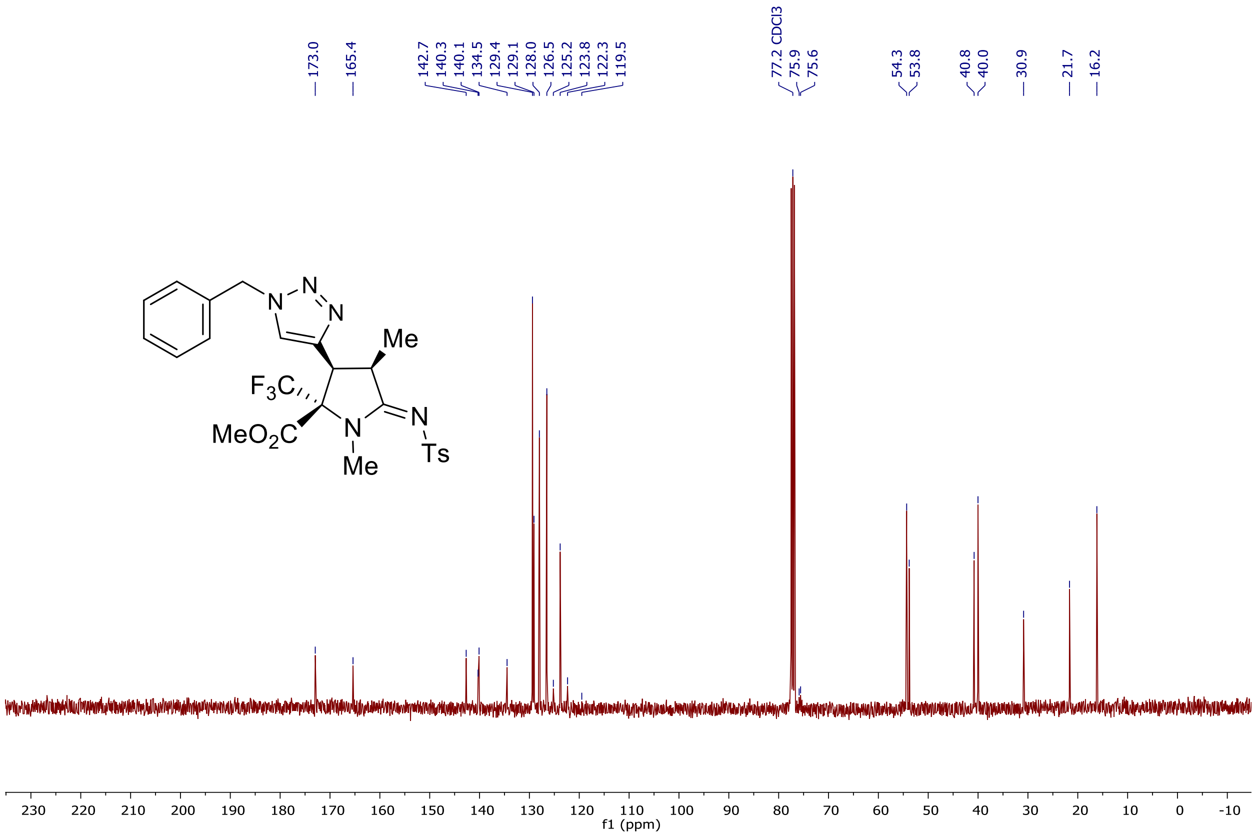

Figure S16. ^{13}C NMR spectrum of **5c** in $CDCl_3$

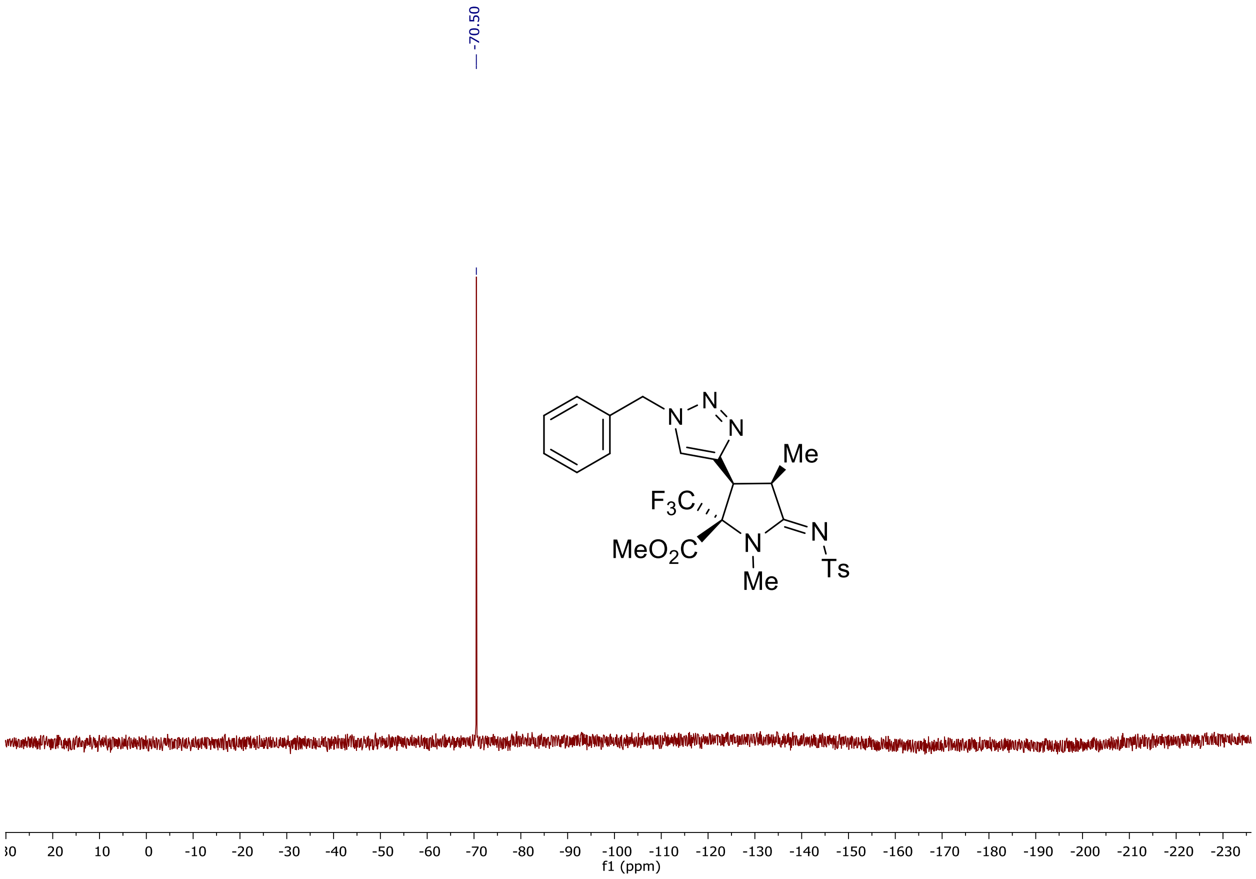

Figure S17. ^{19}F NMR spectrum of **5c** in $CDCl_3$

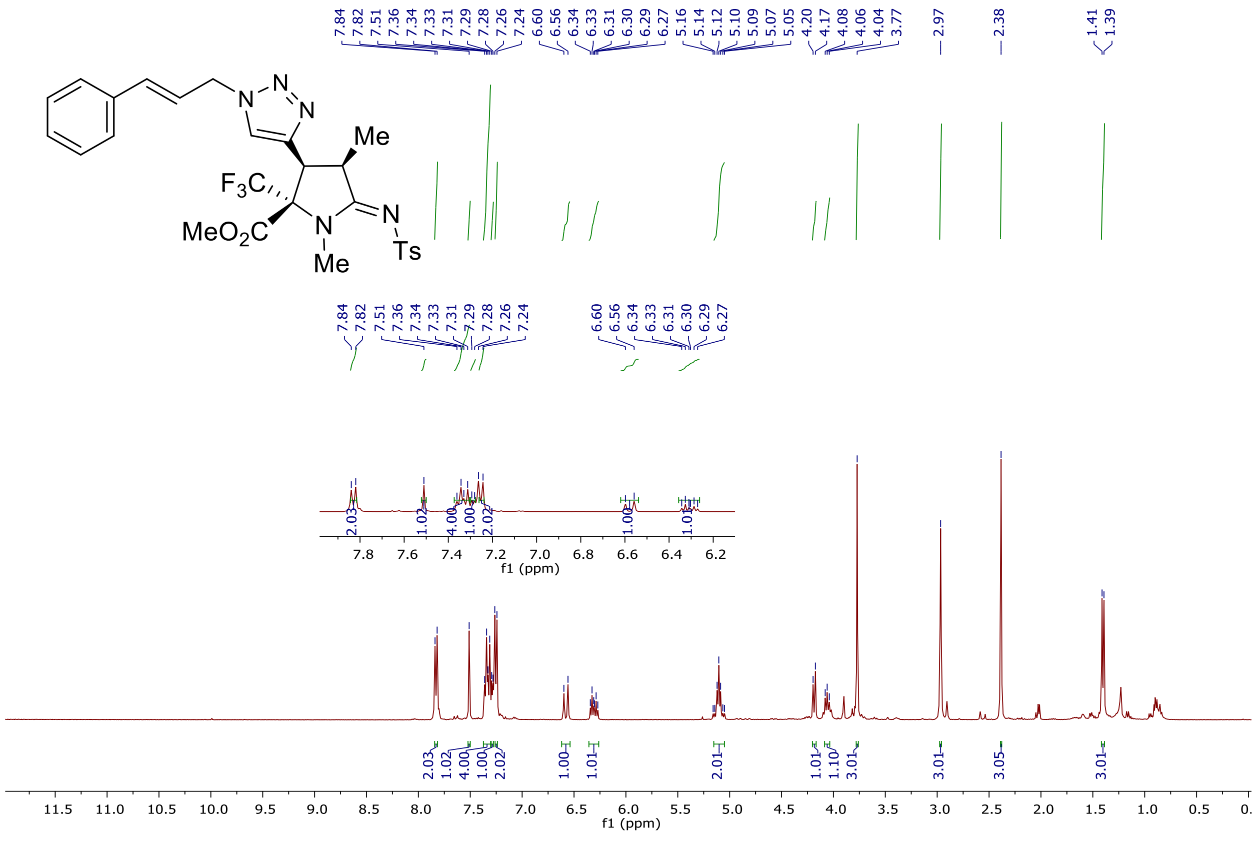

Figure S18. ^{1}H NMR spectrum of **5d** in $CDCl_3$

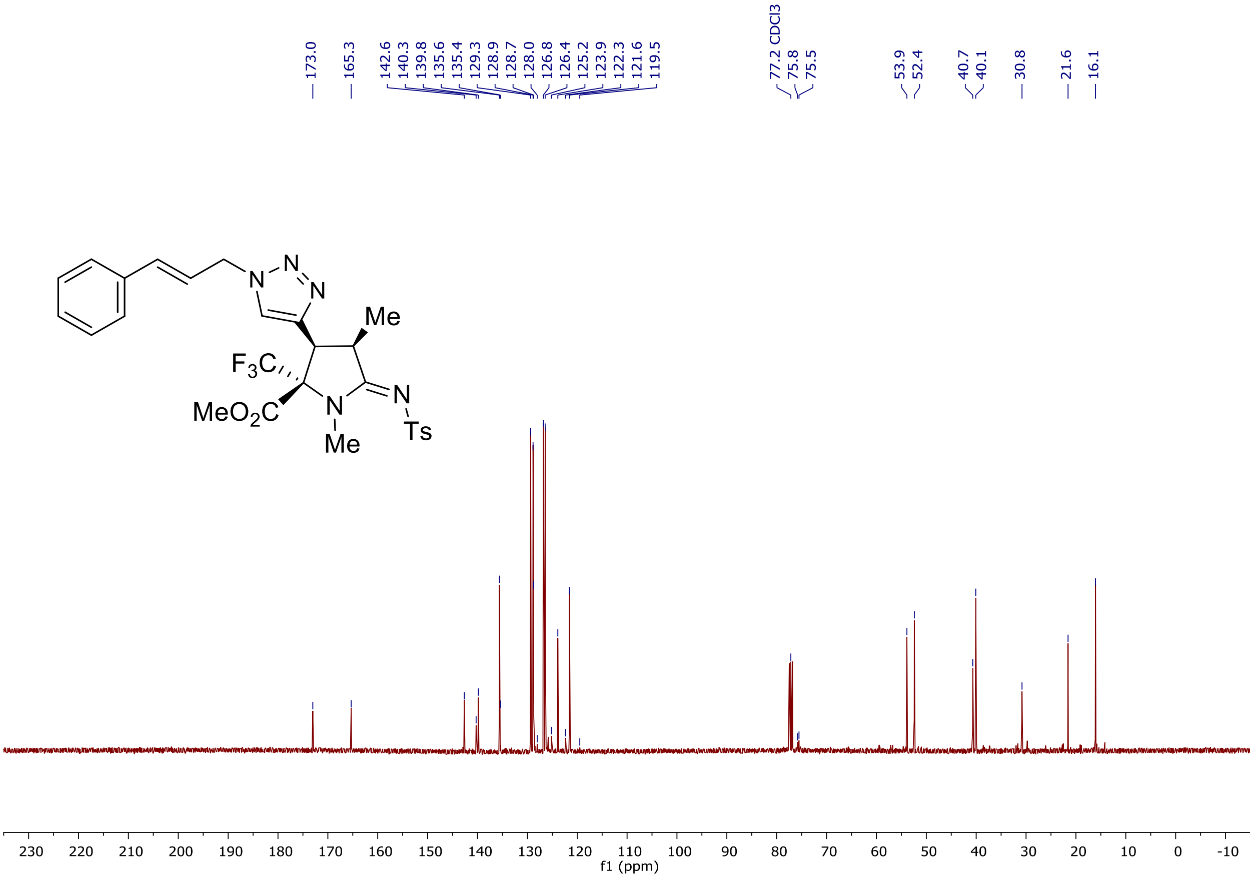

Figure S19. ^{13}C NMR spectrum of **5d** in $CDCl_3$

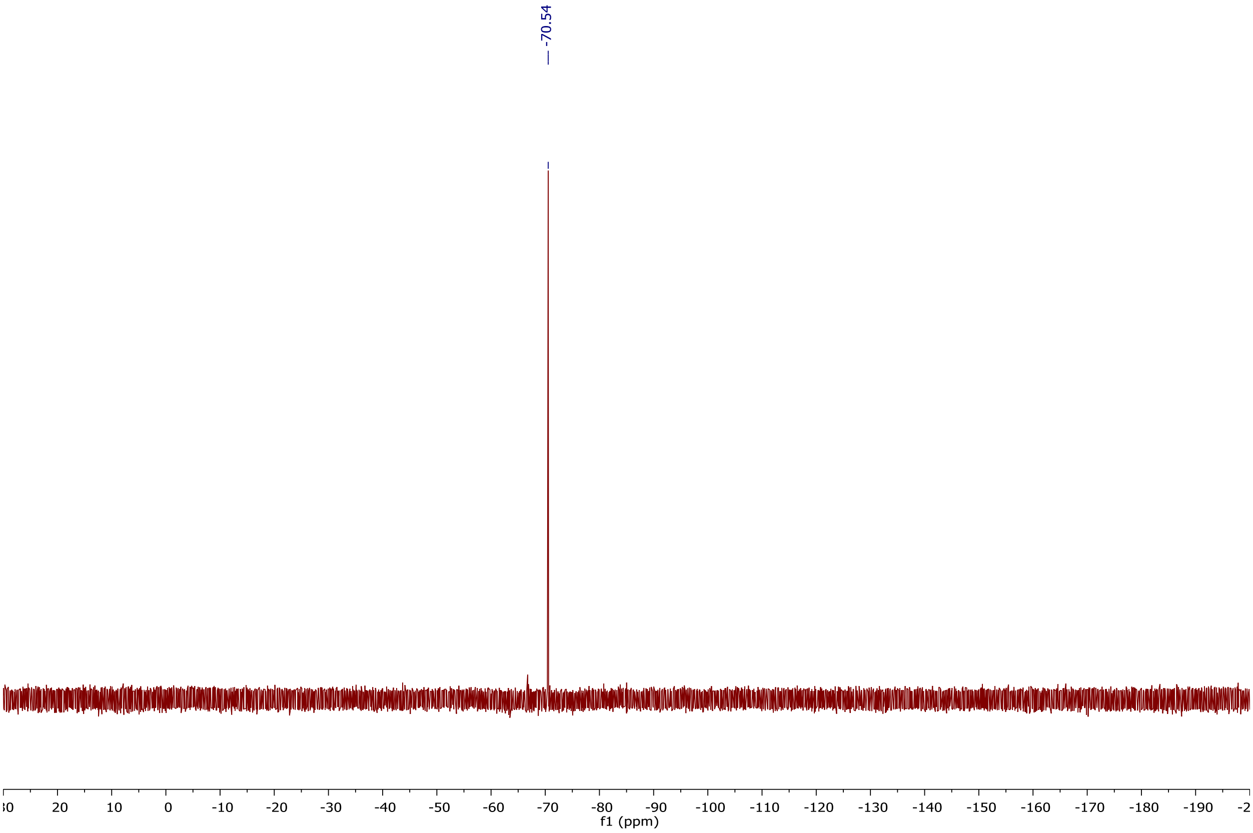

Figure S20. ^{19}F NMR spectrum of **5d** in $CDCl_3$

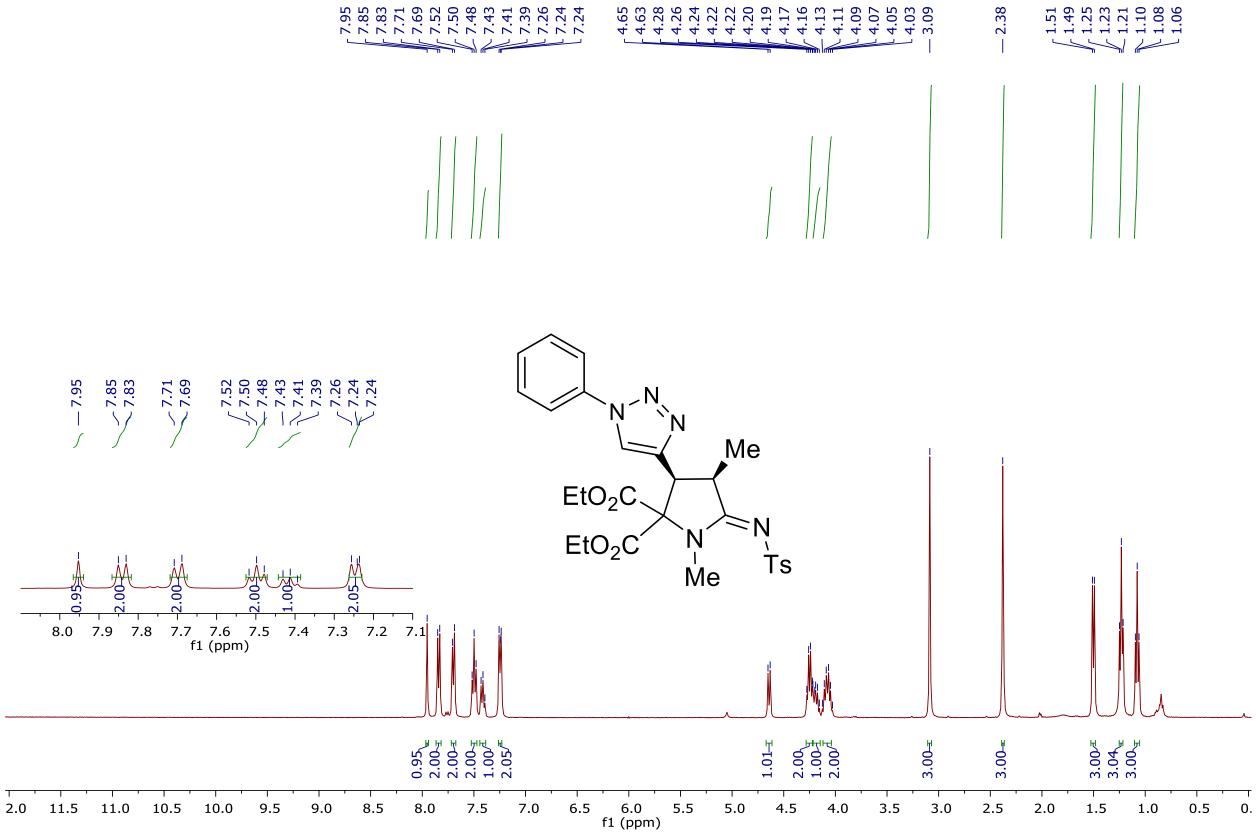

Figure S21. ^{1}H NMR spectrum of **6a** in $CDCl_3$

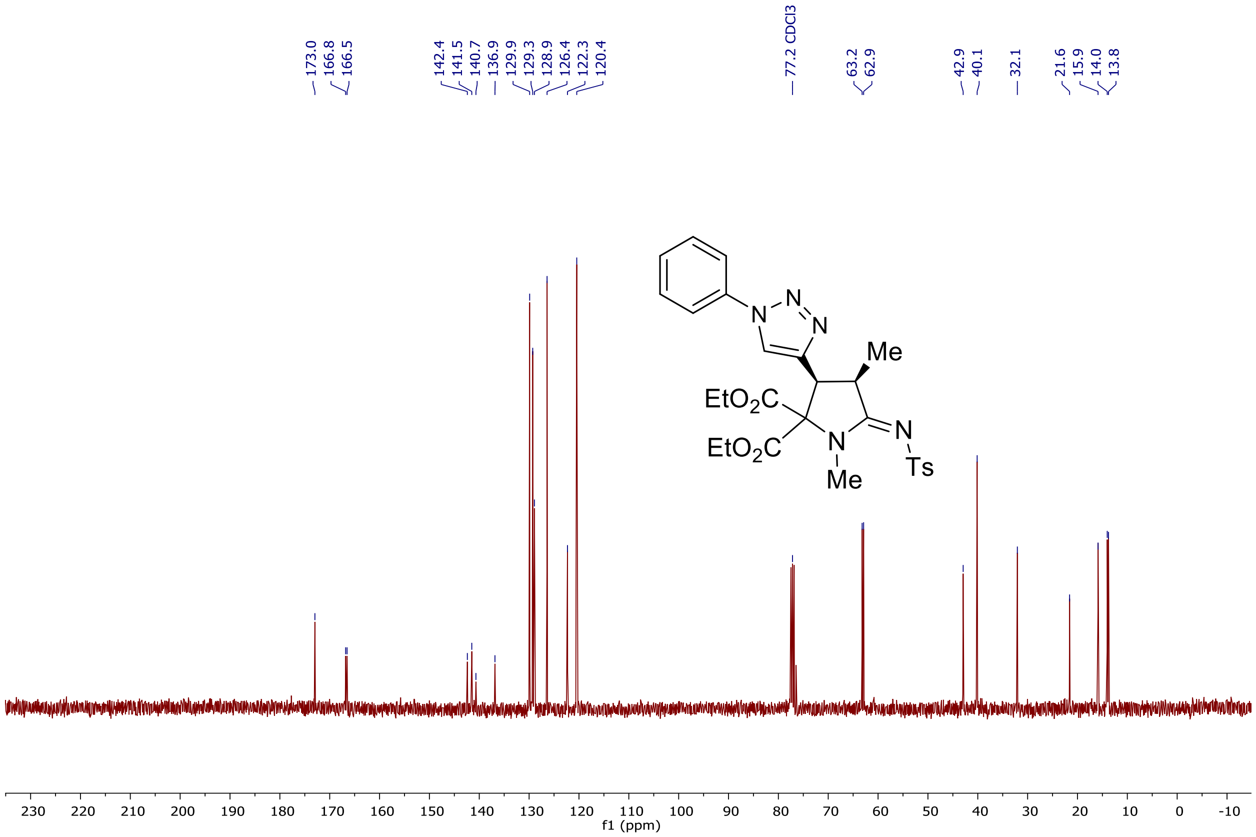

Figure S22. ^{13}C NMR spectrum of **6a** in $CDCl_3$

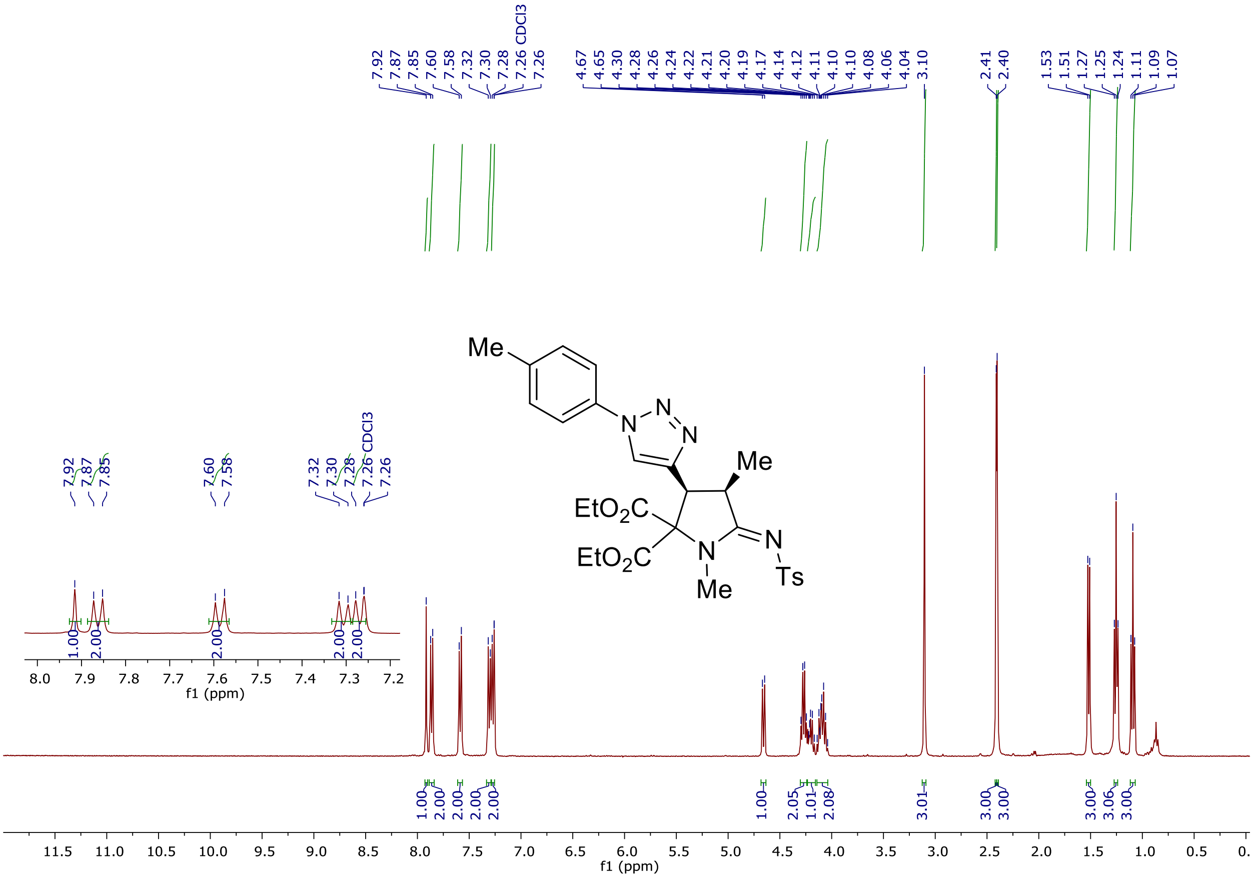

Figure S23. ^{1}H NMR spectrum of **6b** in $CDCl_3$

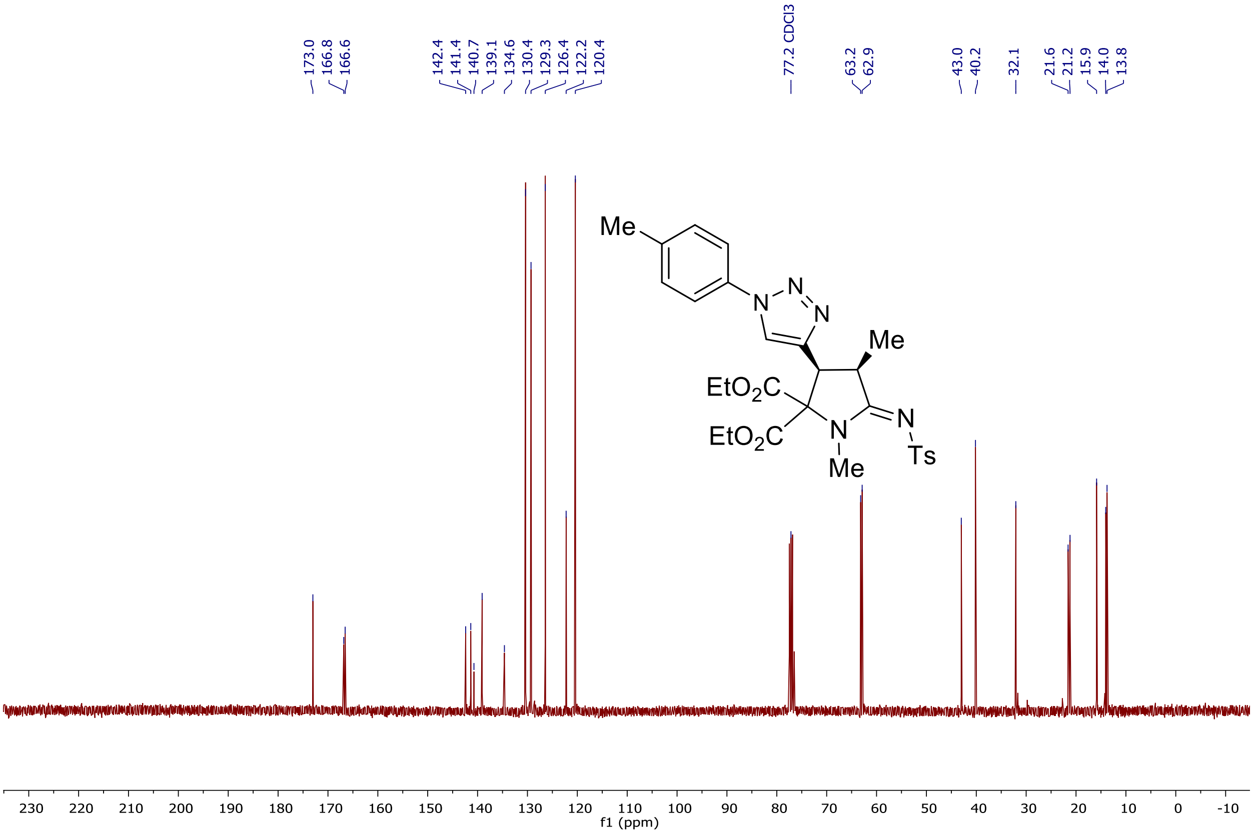

Figure S24. ^{13}C NMR spectrum of **6b** in $CDCl_3$

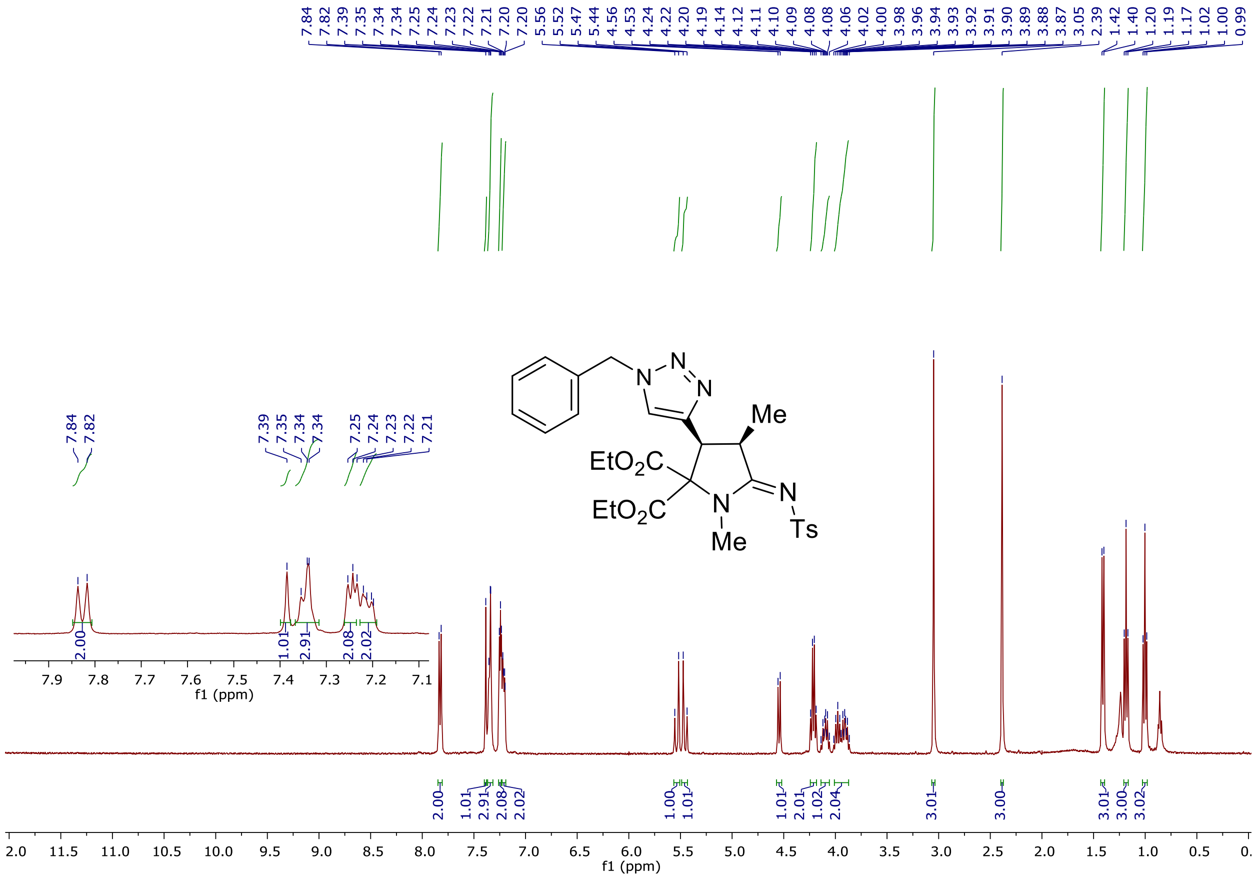

Figure S25. ^{1}H NMR spectrum of **6c** in $CDCl_3$

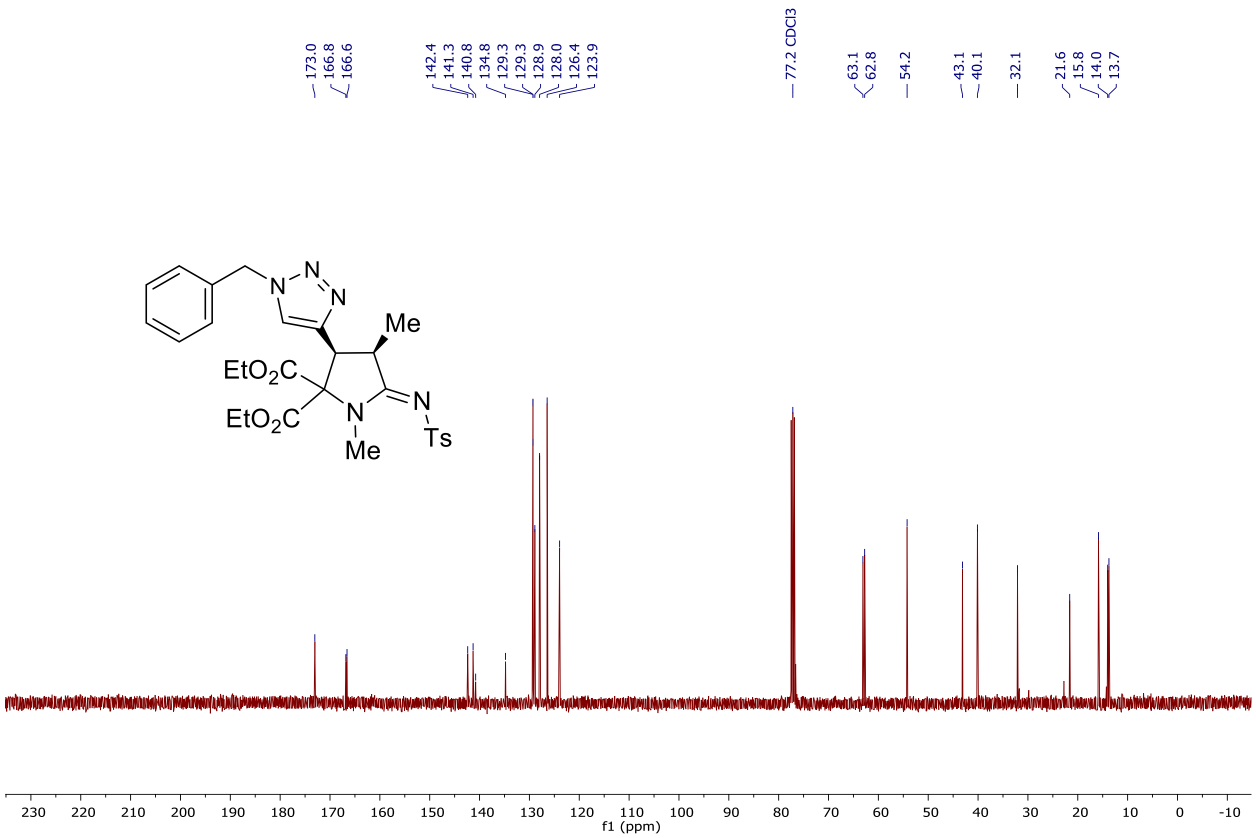

Figure S26. ^{13}C NMR spectrum of **6c** in $CDCl_3$
